# Knockin’ on heaven’s door: Molecular mechanisms of neuronal tau uptake

**DOI:** 10.1111/jnc.15144

**Published:** 2020-09-01

**Authors:** Samantha De La‐Rocque, Edoardo Moretto, Ioana Butnaru, Giampietro Schiavo

**Affiliations:** ^1^ UK Dementia Research Institute University College London London UK; ^2^ Department of Neuromuscular Diseases UCL Queen Square Institute of Neurology University College London London UK

**Keywords:** Alzheimer's disease, endocytosis, intracellular sorting, neurodegeneration, tau, tauopathies

## Abstract

Since aggregates of the microtubule‐binding protein tau were found to be the main component of neurofibrillary tangles more than 30 years ago, their contribution to neurodegeneration in Alzheimer's disease (AD) and tauopathies has become well established. Recent work shows that both tau load and its distribution in the brain of AD patients correlate with cognitive decline more closely compared to amyloid plaque deposition. In addition, the amyloid cascade hypothesis has been recently challenged because of disappointing results of clinical trials designed to treat AD by reducing beta‐amyloid levels, thus fuelling a renewed interest in tau. There is now robust evidence to indicate that tau pathology can spread within the central nervous system via a prion‐like mechanism following a stereotypical pattern, which can be explained by the trans‐synaptic inter‐neuronal transfer of pathological tau. In the receiving neuron, tau has been shown to take multiple routes of internalisation, which are partially dependent on its conformation and aggregation status. Here, we review the emerging mechanisms proposed for the uptake of extracellular tau in neurons and the requirements for the propagation of its pathological conformers, addressing how they gain access to physiological tau monomers in the cytosol. Furthermore, we highlight some of the key mechanistic gaps of the field, which urgently need to be addressed to expand our understanding of tau propagation and lead to the identification of new therapeutic strategies for tauopathies.

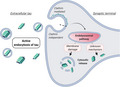

## Graphical abstract

The pathological spread of tau in Alzheimer's disease and other tauopathies follows a stereotypical pattern in the brain, proposed to occur via a prion‐like trans‐synaptic transfer. However, the molecular machinery responsible for the inter‐neuronal uptake of tau has yet to be fully elucidated. This review describes the variety of tau species found within the extracellular space and explores the current proposed mechanisms for tau internalisation, addressing whether they are conserved at the synapse. Finally, it discusses the current understanding of pathological tau release into the cytosol from the endolysosomal pathway, and highlights the main open questions in the field.

**Abbreviations**
AAV: adeno‐associated virus
AD: Alzheimer’s disease
AGD: agyrophilic grain disease
AMPA: α‐amino‐3‐hydroxy‐5‐methyl‐4‐isoxazoleproprionic acid
ApoE: apolipoprotein E
APP: amyloid precursor protein
Aβ: beta‐amyloid
BIN1: bridging integrator 1
CBD: corticobasal degeneration
CCP: clathrin‐coated pit
CCV: clathrin coated vesicle
CIE: clathrin‐independent endocytosis
CME: clathrin‐mediated endocytosis
CRISPRi: CRISPR interference
CSF: cerebrospinal fluid
DIV: days in vitro
DLB: dementia with Lewy bodies
ESCRT: endosomal sorting complex required for transport
EV: extracellular vesicle
FTD: frontotemporal dementia
GlcA: glucuronic acid
GlcNAc: N‐acetyl‐D‐glucosamine
GPI: glycosylphosphatidylinositol
HMW: high molecular weight
HS: heparan sulphate
HSPG: heparan sulphate proteoglycan
iPSC: induced pluripotent stem cell
ISF: interstitial fluid
LDLR: low‐density lipoprotein receptor
LMW: low molecular weight
LRP1: low‐density lipoprotein‐receptor related protein 1
LTP: long‐term potentiation
mAChR: muscarinic acetylcholine receptor
MAPT: microtubule associated protein tau
MFC: microfluidic chamber
MTBR: microtubule binding region
MVB: multivesicular body
NDP52: nuclear dot protein 52
NFT: neurofibrillary tangle
PET: positron emission tomography
PSD: post‐synaptic density
PSP: progressive supranuclear palsy
PTM: post‐translational modification
shRNA: small hairpin RNA
siRNA: small interfering RNA
TBI: traumatic brain injury
TNTs: tunnelling nanotubes
USP: unconventional secretion pathway
WT: wild type

## 1 INTRODUCTION

Tau accumulation in intracellular aggregates is a major hallmark of tauopathies, a group of neurodegenerative diseases which includes Alzheimer's disease (AD), frontotemporal dementia (FTD), agyrophilic grain disease, Pick's disease, progressive supranuclear palsy (PSP), corticobasal degeneration, and traumatic brain injury (Goedert, Wischik, Crowther, Walker, & Klug, 1988; Lee, Goedert, & Trojanowski, 2001). The first mutation in its coding gene microtubule associated protein tau (MAPT) was found to be causative of FTD linked with parkinsonism in 1998 (Hutton et al., 1998; Poorkaj et al., 1998). Since then, tau contribution to neurodegeneration has become well established, fuelling its study as a potential target for AD treatment.

**FIGURE 1:**
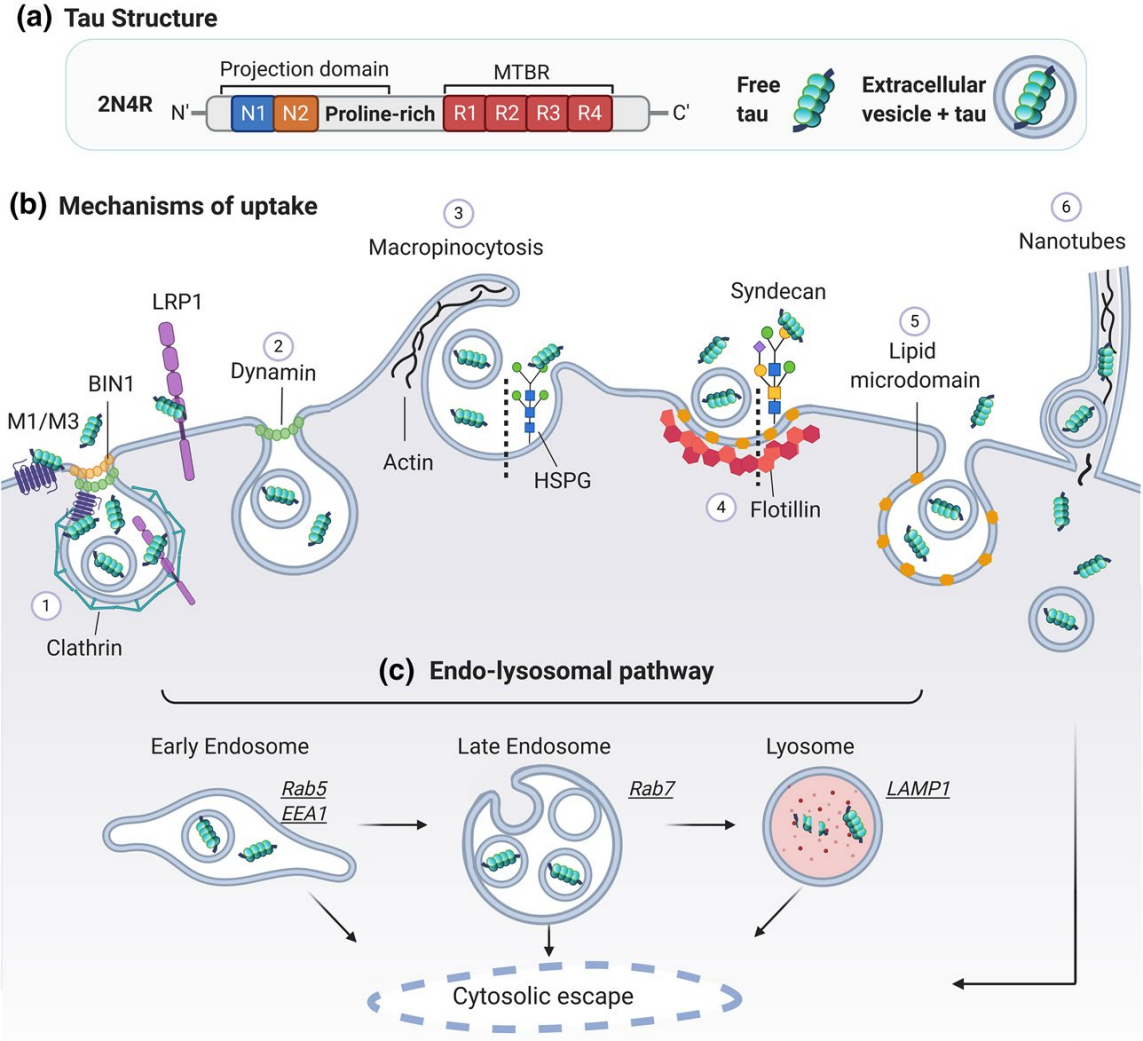
Schematic of tau internalisation mechanisms. (a) Tau structure – Full length 2N4R tau and its functional domains are shown: N‐terminal domain (N1/N2); proline‐rich sequence; microtubule binding region (MTBR) and C‐terminal tail. Tau in the extracellular media can be found as free tau (left) or within extracellular vesicles (right). (b) Mechanisms of tau uptake – (1) Clathrin‐mediated endocytosis (CME) involves the formation of a clathrin‐coated pit. Muscarinic acetylcholine receptor subtype M1 and M3 bind tau at the plasma membrane and are internalised via CME; BIN1 modulates CCP formation and has been involved in tau endocytosis. LRP1 binds tau and the complex is internalised by CME; (2) Dynamin drives membrane fission in both CME and in certain forms of clathrin‐independent endocytosis (CIE); (3) Macropinocytosis is a form of CIE which requires actin‐mediated re‐arrangement of the plasma membrane. Tau binds at the 6‐O sulphation site of heparan sulphate proteoglycan (HSPG), which facilitates tau uptake via macropinocytosis; (4) Lipid microdomains are enriched in cholesterol and sphingolipids, and mediate tau entry by a flotillin‐dependent mechanism. Syndecan is a member of the HSPG family, which binds tau and is internalised via lipid microdomains; (5) Flotillin‐independent lipid microdomain‐mediated uptake; (6) Tunnelling nanotubes have been shown to transport tau; only the recipient cell is displayed. (c) Endo‐lysosomal pathway ‐ Upon internalisation, tau enters the endosomal pathway reaching first early endosomes, which then mature into late endosomes; these can fuse with lysosomes. From these membrane compartments, which have characteristic markers indicated in the panel, internalised tau can be released into the cytosol (see also Figure 2)

The MAPT gene, localised on chromosome 17q21 and composed of 16 exons, generates six isoforms of tau in the human brain (Lee, Cowan, & Kirschner, 1988; Wang & Mandelkow, 2016). Tau is a predominantly unstructured protein (Lee et al., 1988; Schweers, Schönbrunn‐Hanebeck, Marx, & Mandelkow, 1994; Wang & Mandelkow, 2016) and is abundantly expressed in neurons, whereas low levels are found in glial cells (Binder, Frankfurter, & Rebhun, 1985; Shin, Iwaki, Kitamoto, & Tateishi, 1991). Its main known function is to bind and stabilize axonal microtubules through its microtubule binding region (MTBR) (Himmler, Drechsel, Kirschner, & Martin, 1989; Lee, Neve, & Kosik, 1989; Weingarten, Lockwood, Hwo, & Kirschner, 1975). The MTBR is composed of repeats which can be present in 3 or 4 copies (3R or 4R isoforms) depending on the alternative splicing of exon 10. Of note, two short hexapeptide motifs in this region have a tendency to form β‐sheets, a step preceding aggregation (Li & Lee, 2006). The role of the N‐terminal projection domain is less clear, but it is thought to dictate spacing between microtubules (Méphon‐Gaspard et al., 2016). The alternative splicing of exon 2 and 3 leads to the presence of a variable number of repeats generating 0N, 1N or 2N isoforms. Finally, the proline‐rich region contains Thr‐Pro and Ser‐Pro motifs which are targeted by proline‐specific kinases (Wang & Mandelkow, 2016) (Figure 1a). Because of the unfolded nature of tau, several kinases and other modifying enzymes have access to tau generating a large variety of post‐translational modifications (Martin, Latypova, & Terro, 2011; Wang & Mandelkow, 2016).

Under pathological conditions, tau reduces its affinity for microtubules, because of hyper‐phosphorylation (Biernat, Gustke, Drewes, Mandelkow, & Mandelkow, 1993; Bramblett et al., 1993; Drechsel, Hyman, Cobb, & Kirschner, 1992; Yoshida & Ihara, 1993) or mutations (Hong et al., 1998), thus increasing the concentration of free tau to a tipping point, after which its self‐aggregation starts to occur forming oligomers, fibrils and neurofibrillary tangles (NFT) (Braak, Braak, & Mandelkow, 1994). Tau aggregation can also be induced in vitro using the recombinant protein (Crowther, Olesen, Jakes, & Goedert, 1992; Wille, Drewes, Biernat, Mandelkow, & Mandelkow, 1992). Recent work has demonstrated that tau fibrils isolated from different human tauopathies exhibit unique structural properties, showing that tau aggregates are disease‐specific entities (Falcon, Zhang, Murzin, et al., 2018; Falcon, Zhang, Schweighauser, et al., 2018; Falcon et al., 2019; Fitzpatrick et al., 2017; Sanders et al., 2014; Zhang et al., 2019). Some of these differences have recently been proposed to arise from a distinct landscape of post‐translational modifications of tau (Arakhamia et al., 2020).

Pioneering work by Braak and Braak in post‐mortem AD samples (Braak & Braak, 1991), and more recent studies in living patients via positron emission tomography (PET) (Hoenig et al., 2018), demonstrated that tau pathology targets brain regions in a stereotypical temporal sequence correlating with cognitive decline in AD (Braak, Alafuzoff, Arzberger, Kretzschmar, & Tredici, 2006). The evidence that these regions are functionally connected led to the hypothesis that pathological tau spreads from cell to cell similarly to the prion protein (Holmes & Diamond, 2014). In support of this, *in vitro* and *in vivo* work has demonstrated that pathological tau is capable of intercellular transmission and can seed aggregation of endogenous tau (Clavaguera et al., 2009; Frost, Jacks, & Diamond, 2009). Work with aggregated recombinant tau, conditioned media from tauopathy mouse neurons and human cerebrospinal fluid (CSF), has confirmed this prion‐like activity for most tauopathies (Boluda et al., 2015; Clavaguera et al., 2013). The seeding activity of tau has prompted the scientific community to recommend increased vigilance for possible health risks in handling aggregated tau‐containing material in laboratory settings (Lauwers et al., 2020). *In vivo* studies, both by injecting pathological tau in specific brain areas (Clavaguera et al., 2009) and using mouse models with expression of tau restricted to selected brain regions (de Calignon et al., 2012; Liu et al., 2012) indicate that pathological tau spreading is driven by synaptic connectivity and neuronal activity, suggesting its trans‐synaptic transfer (Calafate et al., 2015; Dujardin, Lécolle, et al., 2014; Liu et al., 2012; Wang et al., 2017; Wu et al., 2016).

Although tau aggregation occurs predominantly in neurons, glial cells are also affected in some tauopathies, in particular astrocytes and oligodendrocytes (Kahlson & Colodner, 2015; Lee et al., 2001). Glia over‐activation and secretion of inflammatory cytokines are believed to participate in the induction of tau pathology and neuronal loss in AD [reviewed in (Kahlson & Colodner, 2015; Leyns & Holtzman, 2017)]. Both astrocytes and microglia have been shown to phagocytose soluble and aggregated forms of tau, and to participate in its clearance (Bolós et al., 2016; Luo et al., 2015; Martini‐Stoica et al., 2018; Perea et al., 2019). However, microglia have also been found to re‐exocytose internalized tau within exosomes which could propagate tau pathology to neighbouring neurons (Asai et al., 2015; Hopp et al., 2018). As such, there are crucial roles being discovered for glia in the development and propagation of tau pathology; however, this review will focus on the transfer of tau between neurons in an attempt to decipher the molecular mechanism of its trans‐synaptic spread.

The prion‐like theory hypothesizes a chain of events which would all need to occur for tau pathology to propagate: 1—tau pathology first develops in one neuron; 2—pathological tau is secreted by the ‘donor’ neuron into the extracellular space; 3—a second ‘recipient’ neuron internalizes pathological tau; 4—tau enters the cytosol; and 5—it acts as a seed for endogenous tau in the recipient neuron. Although evidence exists for several of these steps, the exact mechanism is not completely understood.

Here we will summarise the experimental evidence of tau internalisation in the ‘recipient’ neuron, irrespective of its ability to seed aggregation. Because the nature of the tau species has the potential to influence the route of internalisation, we will first describe the diverse forms of tau found in the extracellular space, and then summarise the proposed mechanisms for tau uptake. In addition, we will review the modalities of tau release from the endocytic lumen into the cytosol, finally addressing some of the open questions in the field and suggesting directions for future investigations.

## 2 DIVERSITY OF TAU SPECIES IN THE EXTRACELLULAR MILIEU

Tau secretion from cultured cells, including human induced pluripotent stem cell (iPSC) derived neurons, and mouse models has been reported in both physiological and pathological conditions (Chai, Dage, & Citron, 2012; Mudher et al., 2017; Pérez, Cuadros, Hernández, & Avila, 2016; Pérez, Medina, Hernández, & Avila, 2018). Both full‐length and truncated forms of tau are secreted by neurons (Bright et al., 2015; Kanmert et al., 2015; Karch, Jeng, & Goate, 2013; Plouffe et al., 2012); however, the molecular identity of the released species differs according to the cellular model and the forms of tau being expressed (Mohamed, Herrou, Plouffe, Piperno, & Leclerc, 2013). Similar to cellular and mouse studies, human CSF contains both full‐length (Guix et al., 2018; Wagshal et al., 2015) or fragmented forms of tau (Meredith et al., 2013), with C‐terminal truncated species appearing to be the most abundant. Interestingly, despite the very low levels of full‐length tau identified in human CSF, it was shown via *in vivo* microdialysis that brain interstitial fluid (ISF) from wild‐type (WT) and transgenic 1N4R‐P301S mice contained significantly higher amounts of full‐length tau when compared to CSF (Yamada et al., 2011), suggesting that the absence of full‐length tau observed in some human CSF studies might be because of proteolytic processing in the CSF or lack of detection because of low sensitivity of the assay. RT‐QuIC, a recently developed technology, has enabled the detection of seed‐competent 4R tau in CSF collected from PSP and corticobasal degeneration patients (Saijo et al., 2020). Similarly, CSF collected from post mortem Pick's disease patients was shown to contain seed‐competent 3R‐tau (Saijo et al., 2017). While it is clear that tau secretion occurs in most tauopathies, AD uniquely shows a significant increase in CSF tau levels (Vogels et al., 2020), suggesting that AD evokes an atypical mechanism that might enhance tau secretion.

Extracellular tau has been identified both in its free form and within extracellular vesicles (EVs). EVs are membrane‐enclosed extracellular structures, which can be subdivided into three main subgroups: exosomes, ectosomes (microvesicles) or apoptotic bodies (Mathieu, Martin‐Jaular, Lavieu, & Théry, 2019). While there is no direct evidence of tau release from apoptotic bodies, several reports have observed released tau within ectosomes (Dujardin, Bégard, et al., 2014) and exosomes (Howitt & Hill, 2016; Lee, Mankhong, & Kang, 2019; Polanco, Li, Durisic, Sullivan, & Götz, 2018; Wang et al., 2017). In a study that analyzed the nature of extracellular tau, WT tau was found to be secreted from primary rat cortical cells in a proportion of 90% free tau, 7% in ectosomes and 3% in exosomes (Dujardin, Bégard, et al., 2014), supporting previous findings that free tau is the main extracellular form (Chai et al., 2012; Karch, Jeng, & Goate, 2012).

### 2.1 Free tau

The mechanism of secretion of free tau is still poorly understood. Given that tau lacks a conventional signal peptide (Chai et al., 2012), multiple unconventional secretion pathways (USP) have been proposed. One such USP relies on the direct association between tau and heat shock cognate 70 (Hsc70), in complex with DNAJ heat shock protein family member C5 (DNAJC5) (Fontaine et al., 2015), leading to a SNAP23‐dependent release of tau from secretory endo‐lysosomes (Fontaine et al., 2016). Late endosomal compartments were also involved in a mechanism of tau release modulated by the levels of the small GTPase Rab7A in both primary cortical neurons and HeLa cells (Rodriguez et al., 2017). The suppression of the Golgi‐associated Rab1A was found to increase tau release from both primary cortical neurons and HeLa cells (Mohamed, Desjardins, & Leclerc, 2017). Another proposed mechanism for free tau release is direct translocation across the plasma membrane facilitated by the interaction with PI(4,5)P_2_ and sulphated proteoglycans (Katsinelos et al., 2018; Merezhko et al., 2018). This finding is of particular interest, given the role of heparan sulphate proteoglycans (HSPGs) in tau uptake, as discussed in more detail below.

Secretion of free tau was shown in non‐overexpressing conditions in both primary cortical neurons and SH‐SY5Y neuroblastoma cells (Karch et al., 2012; Mohamed et al., 2013). Upon tau over‐expression, mechanisms of secretion vary according to different cell‐lines used. Transfection of full‐length 2N4R tau in COS‐7 cells led to secretion via exosomes (Simón, García‐García, Gómez‐Ramos, et al., 2012; Simón, García‐García, Royo, Falcón‐Pérez, & Avila, 2012), yet its expression in HEK293T cells caused secretion into the media as free tau (Chai et al., 2012). Similarly, the 0N4R isoform has been shown to be secreted as either free‐tau (Chai et al., 2012; Plouffe et al., 2012), exosomes containing tau (Saman et al., 2012) or within microvesicles (Simón, García‐García, Gómez‐Ramos, et al., 2012; Simón, García‐García, Royo, et al., 2012). Interestingly, truncated tau species appear to be secreted with different efficiencies. Over‐expressed MTBR (tau_250‐370_) was not secreted from COS‐7 cells (Pérez et al., 2016), while over‐expressed C‐terminal truncated tau_1‐421_ was released at significantly higher levels compared to full‐length tau_1‐441_ (Plouffe et al., 2012).

Free tau has been found in the supernatant of cell lines, neurons, ISF of mouse brains and human subject CSF as a mixture of monomers and oligomers; hence, we will discuss evidence for these two forms separately.

#### 2.1.1 Monomeric tau

Extracellular monomeric tau is detected in truncated and hypo‐phosphorylated forms in HeLa cells and rat cortical neurons (Mohamed et al., 2017; Plouffe et al., 2012; Pooler, Phillips, Lau, Noble, & Hanger, 2013). Several tau fragments have been detected both in the extracellular milieu and brain extracts (Quinn, Corbett, Kellett, & Hooper, 2018); in this review we highlight some of the fragments that have been analyzed in more detail. An N‐terminal fragment truncated between amino acids 241/243 has been detected in the supernatant of N2A cells, rat cortical neurons and human iPSC‐derived cortical neurons by ELISA (Kanmert et al., 2015). Similarly, a C‐terminal fragment with propagating properties identified as tau_243‐441_ has been found released by SH‐SY5Y cells (Matsumoto et al., 2015). Moreover tau_243‐441_ levels were increased in brain samples of both transgenic Tg601 mice expressing WT human 2N4R tau and human AD and familial FTD patients (Matsumoto et al., 2015). A different N‐terminal fragment of tau (tau_26‐260_) was detected in the CSF of patients with AD and other dementias (Amadoro et al., 2014) (Table 1). This tau fragment was observed in synapses of cryopreserved human AD brains, and subsequently released in response to depolarization (Sokolow et al., 2015). A recent study identified two abundant populations of tau in the CSF; tau_1‐123_, which is generated during physiological tau processing, and, tau_1‐224_ detected in NFTs and increased in the CSF of AD patients (Cicognola et al., 2019) (Table 1). Importantly, in a study addressing the modulatory role of the N‐terminal elements, 0N isoforms, when over‐expressed, were secreted as N terminal fragments with higher efficiency compared to 1N isoforms in M1C and NB2a/d1 cells (Kim, Lee, & Hall, 2010). Moreover tau cleaved at position 368 by asparagine endopeptidase was found up‐regulated in AD and associated with NFTs (Table 1). Interestingly, the ratio in the CSF between tau_1‐368_ and total tau correlated with tau pathology (Blennow et al., 2020).

**TABLE 1:** Major tau post‐translational modifications detected in the extracellular space

PTM	Tau status	Aggregation status	Detected in	References
Phosphorylation	pT181	Monomers/ oligomers	AD CSF, AD CSF exosomes, AD plasma exosomes, rTg4510 exosomes	Fagan et al. (2007) Mori et al. (1995) Fiandaca et al. (2015) Polanco et al.(2016) Crotti et al. (2019) Barthélemy et al.(2020)
	pS199	HMW	AD brain extract	Takeda et al. (2015)
	pS202	Soluble	AD CSF	Barthélemy et al. (2020)
	pT205			
	pT217			
	pS262	Monomers/oligomers	AD CSF exosomes	Wang et al. (2017)
	pS396/pS404	HMW	AD brain extract, AD CSF, rTg4510 CSF	Takeda et al. (2015) Fiandaca et al.(2015) Barthélemy et al.(2020)

PTM	Tau status	Generated by	Detected in	References
Proteolytic cleavage	aa 1–123	–	AD CSF	Cicognola et al.(2019)
	aa 1–224			
	aa 1−241/243	Calpain 1	N2A cells, rat primary hippocampal neurons, human iPSC derived cortical neurons	Kanmert et al.(2015) Matsumoto et al.(2015)
	aa 1–368	Asparagine endopeptidase	AD CSF	Zhang et al. (2014) Blennow et al.(2020)
	aa 1–402	Caspase 6	AD CSF	Ramcharitar et al. (2013)
	aa 1–421	Caspase 1, 3, 6, 7 and 8	HeLa cells	Plouffe et al. (2012) Gamblin et al.(2003)
	aa 26–230	Caspase 3, Calpain 1 and Calpain 2	AD and other dementias CSF	Park and Ferreira,(2005) Amadoro et al.(2006) Garg, Timm, Mandelkow, Mandelkow, and Wang, (2011) Amadoro et al.(2014) Sokolow et al.(2015)
	aa 243–441	Calpain 1	Tg601 mice brains, AD and familial FTD	Matsumoto et al. (2015)

Although extracellular tau appears to be hypo‐phosphorylated, several phospho sites have good discriminatory power between AD patients and healthy subjects. For example, residue T181 is consistently more phosphorylated in the CSF of AD patients compared to healthy subjects (Fagan et al., 2007; Mori et al., 1995) (Table 1). Interestingly, this phenomenon is uniquely observed in AD and not in other tauopathies (Hampel et al., 2004; Hu et al., 2013). More recently, a longitudinal study identified different phosphorylation states of tau in the CSF of AD patients (Barthélemy et al., 2020) (Table 1). In dominantly inherited AD, the levels of phosphorylation of S202 remained largely constant throughout disease progression, while pT217 and pT181 levels increased during the initial stages of AD and were positively correlated with the initiation of beta‐amyloid (Aβ) pathology and neuronal dysfunction. This was followed by an increase in the levels of pT205 together with soluble total tau (Barthélemy et al., 2020). Levels of pT217 and pT181 were found to decrease at the onset of neurodegeneration, which coincided with the beginning of NFT formation (Barthélemy et al., 2020).

#### 2.1.2 Aggregated tau

Given the importance of tau aggregation in tauopathies, it is not surprising that different forms of tau aggregates are secreted and potentially contribute to transmission of pathology.

In HEK293 cells, over‐expression of the MTBR domain induced the formation of intracellular fibrillar aggregates that were released into the media (Kfoury, Holmes, Jiang, Holtzman, & Diamond, 2012). In the same cell line, over‐expression of the leucine‐rich repeat kinase 2 (LRRK2) promoted the formation and secretion of high molecular weight (HMW) tau species and fibrils (Guerreiro et al., 2016). Furthermore, HEK293 cells and mouse primary cortical neurons over‐expressing the 2N4R isoform were found to generate soluble tau dimers and oligomers that were released into the extracellular medium more efficiently than monomeric tau (Wegmann, Nicholls, Takeda, Fan, & Hyman, 2016). Similarly, in N2A cells over‐expressing 0N4R, 80% of secreted tau was found to be a mix of dimers, trimers and tetramers (Merezhko et al., 2018). These small oligomers are also detected in CSF from AD patients (Sengupta et al., 2017). Interestingly, a protein fragment complementation assay has suggested that, in N2A cells, 99% of tau oligomers are secreted in a vesicle‐free form (Merezhko et al., 2018).

Larger oligomers of tau are also thought to be responsible for tau propagation. A HMW oligomeric form of tau that could transfer between mouse primary cortical neurons was found in brain extracts of transgenic mice including rTg4510 expressing the human 0N4R‐P301L mutant tau and rTg21221 expressing WT 0N4R tau, and in human AD brains (Takeda et al., 2015). This study revealed that the total amount of PBS‐soluble HMW tau was equivalent between AD and healthy brain extracts, however the level of phosphorylation was significantly higher at S199, S396, and S404 sites in AD brains (Takeda et al., 2015) (Table 1). Importantly, similar HMW tau species were identified in ISF and CSF from rTg4510 mice, and in human postmortem ventricular CSF and lumbar CSF from AD patients (Takeda et al., 2016). In particular, a matching phosphorylation site was identified between AD brains and CSF (pS396) (Takeda et al., 2016), suggesting its importance in tau pathology.

### 2.2 Vesicular tau

Despite reports suggesting that 90% of extracellular tau is in free form, it is still not clear which tau species spreads in tauopathies, raising the possibility that vesicular tau could contribute to disease pathogenesis. As previously mentioned, the most relevant EVs implicated in tau transmission are ectosomes and exosomes. Ectosomes are highly heterogeneous EVs that typically range between 100 and 500 nm in diameter, and bud directly from the plasma membrane (Kowal et al., 2016; Meldolesi, 2018). Ectosome‐mediated tau transfer has not been investigated in detail; the only available report demonstrated that when tau is expressed at physiological levels, 7% of released tau is present in ectosomes as full length protein and proteolytic fragments (Dujardin, Bégard, et al., 2014).

In contrast, compelling evidence supports the role of exosomes in the neuronal transfer of tau. Exosomes are single membrane vesicles with a diameter between 50 and 150 nm (Mathieu et al., 2019). They originate from multivesicular bodies (MVB), containing multiple intraluminal vesicles (ILVs) of endosomal origin (Simons & Raposo, 2009). MVBs can fuse with the plasma membrane, releasing their ILVs into the extracellular space, which are classified as exosomes (Mathivanan, Ji, & Simpson, 2010). The mechanism of exosome release is dependent on synaptic activity (Bahrini, Song, Diez, & Hanayama, 2015; Lachenal et al., 2011) and forms a major pathway for intercellular communication (Pegtel & Gould, 2019). Exosomes activate surface receptors and deliver cargoes, including proteins, lipids, mRNA, miRNA and non‐coding RNAs, to neighbouring cells. They have previously been involved in the intercellular transfer of pathological proteins, such as α‐synuclein, prion protein and Aβ (Howitt & Hill, 2016).

Seed‐competent monomeric and oligomeric (trimeric) tau phosphorylated at S262, S396 and/or S404 (Otvos et al., 1994) was identified in exosomes isolated from CSF of AD patients (Wang et al., 2017) (Table 1). Exosomes of brain origin have also been found in plasma of AD patients; when compared to controls, AD exosomes contained higher levels of total, pT181‐ and pS396‐tau. Importantly, these post‐translational modifications (PTMs) were able to predict disease development ten years prior to AD diagnosis (Fiandaca et al., 2015).

While tau present in exosomes isolated from rTg4510 mice had the highest phosphorylation levels at T181, other sites associated with NFTs (e.g. S202/T205, T212/S214 and T231), were less phosphorylated, suggesting that exosomes carry a low amount of aggregated tau (Polanco, Scicluna, Hill, & Götz, 2016). Recently, exosomes purified from AD CSF containing seed competent pT181‐tau were found to be positive for Bridging integrator 1 (BIN1) (Crotti et al., 2019), a protein also involved in tau endocytosis. Interestingly, over‐expression of BIN1 in HEK293T cells was shown to increase 2N4R‐tau release in exosomes (Crotti et al., 2019).

## 3 EVIDENCE FOR TRANS‐SYNAPTIC SPREAD OF TAU

Several lines of evidence suggest that tau pathology spreads via a trans‐synaptic mechanism as demonstrated by experiments in primary neurons grown in microfluidic chambers (MFCs) (Calafate et al., 2015; Dujardin, Lécolle, et al., 2014; Hallinan, Vargas‐Caballero, West, & Deinhardt, 2019; Nobuhara et al., 2017; Takeda et al., 2015; Wang et al., 2017). In these devices, neurons are grown into chambers connected through microgrooves that allow only axons to pass through, thus modelling a neuronal circuit. In addition, MFCs allow control over the direction of media flow between chambers, thus making it possible to assess whether transfer of material between cells is mediated by direct cell contact. In a three‐chamber MFC, the absence of neurons in the middle chamber prevents the transfer of tau between neurons plated in the first and third chamber; this was observed upon incubation with tau‐containing exosomes isolated from N2A cells (Wang et al., 2017) and in tau‐transduced neurons seeded with aggregated tau (Calafate et al., 2015). Since axon terminals that passed into the middle chamber could still secrete tau, this result demonstrates that close neuronal contact is needed for efficient tau uptake.

A further validation of the trans‐synaptic spreading of tau came from the observation that the knockdown of the post‐synaptic adhesion proteins neuroligin‐1, −2 and −3, which are crucial for synapse formation and maturation (Südhof, 2018), strongly reduced transfer of aggregated tau in MFCs (Calafate et al., 2015). Independent work has supported the requirement of mature synapses to enable tau uptake, since no transfer of tau‐containing exosomes occurred between mature presynaptic neurons connected with immature post‐synaptic neurons (DIV4) where no synapses were observed (Wang et al., 2017). However, abnormalities in post‐synaptic architecture impinge on the maturation of the pre‐synaptic compartment (Südhof, 2018); thus, the absence of tau transfer could also be because of an impaired pre‐synaptic release of tau. These conclusions are controversial, since an independent study has shown that over‐expressed WT tau could transfer within MFCs using DIV2 receiving neurons (Dujardin, Lécolle, et al., 2014). This might suggest that synaptic contact is required for the transmission of pathological but not physiological tau.

The importance of synaptic connections in tau spread *in vivo* has been observed using different experimental systems, including injection of tau aggregates (Ahmed et al., 2014; Clavaguera et al., 2009), transgenic mice expressing tau only in the entorhinal cortex (de Calignon et al., 2012; Liu et al., 2012) and using viruses to restrict tau expression in specific brain regions (Dujardin, Lécolle, et al., 2014; Wegmann et al., 2019). In all cases, tau spread was observed with greater efficiency to areas that are functionally connected with the donor region. Although most of the work shows spread of tau from the pre‐ to the post synapse, some studies revealed potential transmission in the opposite direction. For example, Wu and colleagues observed that low molecular weight (LMW) recombinant tau aggregates can be taken up by the axonal compartment in primary neurons (Wu et al., 2013). Similarly, *in vivo* experiments suggested spread of tau in a ‘retrograde’ direction (Ahmed et al., 2014). Studies based on tau PET of AD patients coupled with mathematical predictions modelling tau spread in the human brain, have also supported the importance of neuronal connectivity in this process (Alzheimer’s Disease Neuroimaging Initiative et al., 2020; Franzmeier, Rubinski, Neitzel, Kim, et al., 2019; Hoenig et al., 2018; Jacobs et al., 2018).

To pharmacologically target the transmission of tau at synapses for clinical benefit, a greater understanding of the molecular and cellular mechanisms of tau uptake is required. Next, we will discuss the proposed mechanisms for the cellular entry of tau and address whether they are conserved at the synapse.

## 4 MECHANISMS FOR NEURONAL ENTRY OF TAU

### 4.1 Endocytic mechanisms of uptake

Understanding the molecular machinery mediating the cellular uptake of tau has been a priority of the field since the emergence of the prion‐like hypothesis in tauopathies. Both monomeric and aggregated forms of tau can be internalised via a temperature‐dependent process, suggesting active endocytosis (Frost et al., 2009; Michel et al., 2014). In addition, tau co‐localises with intracellular vesicular markers upon internalisation (Michel et al., 2014). Since several groups have ruled out direct membrane translocation (Frost et al., 2009; Holmes et al., 2013), in this chapter we discuss four proposed mechanisms for tau internalisation: clathrin‐mediated endocytosis (CME), clathrin‐independent endocytosis (CIE), EV uptake and tunnelling nanotubes (Table 2). However, given multiple forms of cell‐secreted tau are present in the extracellular medium, it is unlikely that one single mechanism of internalisation will account for all forms of cell‐to‐cell transmission.

**TABLE 2:** Summary of findings for key tau internalisation mechanisms

Uptake mechanism	Model	Aggregation status/ Isoform/ Source	Method of investigation	Key findings
Clathrin mediated endocytosis				
Clathrin‐dependent Falcon, Noad, et al. (2018)	HEK293 and SH‐SY5Y cells	Monomeric/ 0N4R‐P301S/ recombinant	Expression of dominant negative forms of endocytic components Chemical inhibitors: Actin‐ latrunculin A and EIPA; CDC42‐ ZCL278; PI3K‐ wortmannin and LY2940002	Actin, dynamin, CDC42, PI3K, AP180, FCH02 are involved in tau uptake
BIN1 associated Calafate et al.(2016)	Primary hippocampal neurons expressing HA−2N4R‐P301L	Aggregated/ 2N4R‐P30L/ HEK293 cell secreted	Chemical inhibitors: Dynamin‐ Dynasore and Dynamin JNJ BIN1 knockdown and BINV1 over‐expression	BIN1 activity reduces tau uptake, measured by tau aggregation Dynamin role
Muscarinic AChR mediated Morozova et al. (2019)	HEK293 and CHO cells Primary cerebellar neurons	Not specified/ 2N4R‐R406W and phosphomimetic/ recombinant	Cell line and neurons: chemical inhibitors: mAChR‐ atropine M1mAChR ‐ pirenzipine Cell line: exogenous expression of mAChRs	Cell line and neurons: mAChR activation promotes tau uptake Cell line: M1/M3 mAChR subtypes promote tau uptake
LRP1 mediated Rauch et al. (2020)	H4 cells Human iPSC‐derived neurons WT mouse injected with AAV‐EGFP−2A−2N4R P301L	Monomeric/ 0N4R,1N4R, 2N4R, 0N3R, 1N3R, 2N3R and MTBR; oligomeric/ 2N4R; sonicated fibrils/ 2N4R; recombinant	Cell line and neurons: CRISPRi mediated LRP1 knockdown *In vivo* *:* shRNA‐mediated knockdown of LRP1	Cell line and neurons: LRP1 mediates uptake of monomeric and aggregated tau *in vivo*; LRP1 participates in 2N4R‐P301L spread
Clathrin independent endocytosis				
Clathrin independent Falcon, Noad, et al. (2018)	HEK293 and SH‐SY5Y cells	Monomeric/ 0N4R‐P301S/ recombinant	See CME: Falcon, Noad, et al. (2018)	Actin, dynamin, CDC42 and PI3K dependency
Bulk/fluid phase endocytosis; Wu et al. (2013)	HeLa cells Primary cortical and hippocampal neurons in microfluidic chambers	LMW aggregates and sonicated short fibrils/ 2N4R/ recombinant	Neurons: Chemical inhibitor: Dynamin‐ dynasore	Cell line and neurons: dextran co‐localisation Neurons: dynamin‐ dependency Neurons: Aggregates uptake at pre‐ and post‐synapse
Macropinocytosis and dynamin dependent Evans et al. (2018)	Human iPSC‐derived cortical neurons	Monomeric and aggregated/ 2N4R‐P301S/ recombinant	Chemical inhibitors: Dynamin‐ dynasore; Actin‐ cytochalasin D	Monomeric tau: Dynamin dependency (Rapid, short‐lived) Actin dependency Aggregated tau: Dynamin dependency (sustained inhibition) Actin independent
HSPG; Macropinocytosis Holmes et al.(2013)	C17.2 cells Primary hippocampal neurons WT mice	Sonicated MTBR and fibrils/ 2N4R/ recombinant	Cell line: Chemical inhibitors: Dynamin‐ dynasore; Actin‐ cytocholasin D and latrunculin; Cell line and neurons: Heparin treatment, HSPG sulphation inhibitor – sodium chlorate *In vivo*: injection of heparin mimetic F6	Cell line: Actin dependency/ dynamin independent cell line and neurons: Heparin sensitivity *in vivo:* F6 reduced 2N4R fibril uptake in cortical neurons
HSPG; Macro‐pinocytosis Puangmalai et al.(2020)	Primary cortical neurons	Tau oligomers / not specified/ AD, PSP and Dementia Lewy Body post‐mortem human brain	Chemical inhibitors: Dynamin‐ dynasore; Caevolin‐ nystatin Heparin treatment HSPG biosynthesis: knockdown of Ext2	Dynamin and caveolin independent Dextran co‐localisation Heparin sensitivity HSPG formation required for tau uptake
HSPG; Role of Heparin sulphation 1. *Stopscinski et al* (2018) 2. * Rauch et al.(2018)	HEK293 and C17.2 cells	Sonicated fibrils/ 2N4R/ recombinant	Cell line: CRISPRi‐knockdown of HSPG biosynthesis genes *In vitro*: tau binding heparin derivatives Heparin and derivatives treatment *Cell line: Chemical inhibitor: Dynamin‐ dynasore	1. *in vitro*: Tau binds 6‐O and *N*‐sulphated HSPGs tau uptake requires: HSPG formation/ sulphation
	H4 cells Human IPSC‐derived neurons Adult mouse brain slices	Monomeric, oligomeric and sonicated fibrils/ 2N4R/ recombinant		2. *in vitro*: Tau binds 6‐O‐sulphated HSPG Cell line/ neurons/ ex vivo: tau uptake requires HSPG formation and sulphation *Cell line: dynamin dependency
HSPG: Syndecans Hudák et al. (2019)	K562 and SH‐SY5Y cells	Monomeric and sonicated fibrils/ 2N4R/ recombinant	Exogenous expression of syndecan1−4 Chemical inhibitor: HSPG sulphation‐ sodium chlorate Co‐IP of lipid microdomains	Syndecans 2–4 mediate uptake Syndecans associate with flotillin1−2 Monomeric tau aggregation before uptake
Extracellular vesicular (EV)‐tau uptake				
EV‐mediated 1. Polanco et al.(2016) 2. Polanco et al.(2018)	FRET Biosensor HEK293 cells	EV tau/ 0N4R‐P301L/ rTg4510 mouse	FRET: Flow cytometry and confocal microscopy	1. Cell line: EV‐tau is internalised and seeds aggregation
	Primary hippocampal neurons		Super‐resolution and electron microscopy	2. Neurons: EV‐tau within soma, dendrites and axons
EV‐mediated Ruan et al. (2020)	Primary cortical neurons WT mice	EV tau and aggregates/ unknown isoform/ post‐mortem human brain from healthy, prodromal and symptomatic AD	FRET: flow cytometry and confocal microscopy	Neurons: Symptomatic AD–EV most efficiently internalised *In vivo:* EV spread more efficiently than free tau
Alternative entry mechanisms				
Tunnelling nanotubes (TNT) mediated Tardivel et al. (2016)	CAD cells Primary cortical neurons transduced with 1N4R	Sonicated fibrils/ 1N4R/ recombinant	Live and fixed samples confocal microscopy	Cell line and neurons: exogenous tau triggers TNT formation TNT contain endogenous and recombinant tau

#### 4.1.1 Clathrin‐mediated endocytosis of tau

##### The role of clathrin, dynamin and BIN1

CME is responsible for the selective internalisation of a wide variety of cargoes, which are typically trafficked via the endo‐lysosomal pathway. CME relies on the formation of a curved clathrin lattice surrounding the budding plasma membrane, which eventually generates a clathrin‐coated pit (CCP). Clathrin aids in stabilising the membrane curvature and in generating the CCP neck, thus facilitating the separation of clathrin‐coated vesicles (CCV) (Doherty & McMahon, 2009), with diameters of 80–120 nm on average (Pearse, 1976). The GTPase dynamin is recruited to the neck of CCP and drives membrane fission, triggering the release of the CCV (Doherty & McMahon, 2009).

For trans‐synaptic transmission of tau to occur, CME events need to happen at pre‐ and/or post‐synaptic sites. CME is an established mechanism at the post‐synapse with most neurotransmitter receptors undergoing CME upon stimulation (Jung & Haucke, 2007). On the presynaptic side, CME is implicated in multiple processes, including the recycling of synaptic vesicles (Jung & Haucke, 2007).

CME has been implicated in the transmission of tau and its intracellular delivery (Figure 1b; 1). Recent work demonstrated that 2N4R‐P301L aggregates secreted by HEK293 cells displayed 40% co‐localisation with the CME marker transferrin when incubated for four hours with rat hippocampal neurons (Calafate, Flavin, Verstreken, & Moechars, 2016). Dynamin‐dependent internalisation of pathological tau was shown in neurons expressing HA‐2N4R‐P301L after application of cell‐secreted 2N4R‐P301L aggregates (Calafate et al., 2016). Incubation of neurons with dynasore, a dynamin inhibitor, caused a reduction, yet not a complete disappearance, of HA‐positive tau aggregates (Calafate et al., 2016). While dynamin activity is not exclusively linked to CME, this evidence suggests that CME may be involved in the uptake of tau aggregates. CME has also been shown to mediate uptake of recombinant monomeric 0N4R‐P301S tau in HEK293 and SH‐SY5Y cells by suppressing the activity of CME components, including FCHO and AP180, by expressing their dominant‐negative forms; interestingly, this mechanism was not shared by recombinant aggregated 0N4R‐P301S tau, which was found to undergo dynamin‐dependent CIE (Falcon, Noad, McMahon, Randow, & Goedert, 2018).

The role of CME in tau internalisation was further explored after BIN1 was linked to AD. BIN1 belongs to a family of BAR domain‐containing proteins and facilitates endocytosis by binding clathrin and dynamin to assist CCP formation and initiate CCV fission (Doherty & McMahon, 2009). Crucially, BIN1 is the second most statistically‐significant genetic locus after apolipoprotein E (ApoE) associated with the development of late onset AD. In particular, single nucleotide polymorphisms of BIN1 confer greater risk of developing AD and correlate with increased tau load, as assessed by PET (Franzmeier, Rubinski, Neitzel, Ewers, and Alzheimer’s Disease Neuroimaging Initiative (ADNI), 2019). Interestingly, elevated levels of BIN1 mRNA are associated with increased tau in both healthy (Franzmeier, Rubinski, Neitzel, & Ewers, 2019) and AD brains (Chapuis et al., 2013). Conversely, BIN1 protein levels have been found to be reduced in AD brains with no associated reduction in tau levels but rather a mis‐sorting of tau to the synapse (Glennon et al., 2020). As such, the nature of the association between BIN1 mRNA and protein levels and tau accumulation remains unclear.

Calafate and colleagues identified a relationship between BIN1 and tau uptake by CME (Calafate et al., 2016) (Figure 1b; 1). Upon BIN1 down‐regulation by shRNA in hippocampal neurons, an approximate 30% increase in HA‐2N4R‐P301L aggregates was observed in a tau propagation assay (Calafate et al., 2016). Consistently, the opposite trend was detected when BIN1V1 (human neuronal isoform 1) was over‐expressed (Calafate et al., 2016).

Upon endocytosis, cargoes are transported to early endosomes, that are associated with the small GTPase Rab5 (Mayor & Pagano, 2007). BIN1 down‐regulation evoked an increase in both number and size of Rab5‐positive puncta (Calafate et al., 2016), which suggests enhanced recruitment of the active form of Rab5 to promote endocytosis (Stenmark et al., 1994). These data predict that reduced levels of BIN1 boosts activated Rab5 recruitment, thus increasing the influx of tau into forming endosomes. This model is supported by the observation of reduced BIN1V1 levels and enlarged endosomes in human AD brains (Cataldo et al., 2000; Holler et al., 2014).

BIN1 is present at pre‐ and post‐synaptic sites and in axons, as shown by super‐resolution microscopy and immuno‐electron microscopy in primary hippocampal neurons and CA3‐CA1 synapses (De Rossi et al., 2020; Schürmann et al., 2019). Post‐synaptically, BIN1 is enriched at extra‐synaptic sites within the mouse cortex (Schürmann et al., 2019); given that CME largely occurs at sites adjacent to the post‐synaptic density (PSD) (Triller & Choquet, 2005), the localisation of BIN1 supports its involvement in the CME of tau at post‐synaptic sites and suggests a role for BIN1 in its trans‐synaptic spread.

##### Receptor‐dependent CME

Further evidence for CME of tau comes from findings pointing to muscarinic acetylcholine receptors (mAChRs) as mediators of tau uptake (Figure 1b; 1). Treatment with atropine, a non‐selective mAChR antagonist, caused an 80% reduction in the uptake of recombinant WT and phosphomimetic 2N4R‐R406W tau (S199, T212, T231, and S262 mutated to glutamate) in HEK293 cells and cerebellar neurons (Morozova et al., 2019). To identify which mAChR subtypes were implicated in the process, selective inhibitors for the M1, M2 and M4 mAChRs were tested. Only the M1‐selective antagonist pirenzepine caused a significant inhibition of WT and phosphomimetic tau uptake. Because selective inhibitors for M3‐mAChRs are currently not available, the authors tested the effects of over‐expression of M1 or M3 subtypes; while this treatment increased 19‐fold the uptake of both WT and phosphomimetic tau, tau uptake increased by 30‐fold when both M1 and M3 were co‐expressed (Morozova et al., 2019). This additive effect supports the hypothesis that both mAChR subtypes are involved in tau uptake.

Interestingly, M1/M3‐mAChRs are internalised via CME both in basal conditions (Lee, Pals‐Rylaarsdam, Benovic, & Hosey, 1998) and upon ligand binding (Lameh et al., 1992). It is therefore possible that mAChRs are directly involved in tau internalisation, since tau can bind to M1/M3‐mAChRs and act as receptor agonists as shown both *in vitro* and *in vivo* (Gómez‐Ramos, Díaz‐Hernández, Rubio, Miras‐Portugal, & Avila, 2008; Martinez‐Aguila et al., 2014). M1 and M3 have been detected at the pre‐ and post‐synaptic sites in the hippocampus (Levey, 1996; Rouse, Gilmor, & Levey, 1998; Rouse & Levey, 1997; Yamasaki, Matsui, & Watanabe, 2010), with M1 mostly detected outside the PSD (Yamasaki et al., 2010). This raises the possibility that mAChRs could mediate trans‐synaptic spread of tau at synaptic sites. This evidence together with the proven, although limited, clinical effect of cholinesterase inhibitors in the treatment of cognitive impairment in AD (Anand & Singh, 2013) strongly support the involvement of mAChRs in tau pathology.

Following a CRISPR interference silencing screening, the low density lipoprotein‐receptor related protein 1 (LRP1) was found to be the only member of the family of low‐density lipoprotein receptors to be involved in tau internalisation (Rauch et al., 2020) (Figure 1b; 1). LRP1 is a large membrane protein that is cleaved into two non‐covalently bound polypeptides; the C‐terminal fragment (85 kDa) is anchored to the plasma membrane, whilst the N‐terminus (515 kDa), composed of four functional domains, is extracellular (Emonard, Théret, Bennasroune, & Dedieu, 2014; Herz & Strickland, 2001). LRP1 has originally been described as the endocytic receptor for α‐2‐macroglobulin, yet over 40 ligands have been identified to date (Bres & Faissner, 2019). The majority of these interactions occur at the level of the extracellular domain II and IV. LRP1 has been involved in several biological pathways, such as regulation of cholesterol homeostasis, intracellular signalling and synaptic integrity (Emonard et al., 2014; Herz & Strickland, 2001).

Many LRP1 ligands have been linked to AD pathology, including ApoE (Rebeck, Reiter, Strickland, & Hyman, 1993), cell‐surface amyloid precursor protein (Knauer, Orlando, & Glabe, 1996) and secreted amyloid precursor protein (Kounnas et al., 1995). In addition, the neuronal processing of Aβ and its clearance via the blood brain barrier are modulated by LRP1 (Fuentealba et al., 2010; Shibata et al., 2000). However, the precise nature of LRP1 involvement in AD pathogenesis is still widely debated (Kanekiyo & Bu, 2014; Shinohara, Tachibana, Kanekiyo, & Bu, 2017); nevertheless, the ability to interact with ApoE‐Aβ complex, and this novel interaction with tau, makes LRP1 an emerging link between major players of AD pathology. Although LRP1 partially localises to lipid microdomains, it is mainly internalised by CME‐dependent mechanisms, as shown for many of its ligands, including prion protein (Jen et al., 2010; Taylor & Hooper, 2007), Aβ (Fuentealba et al., 2010) and CD44 (Perrot et al., 2012).

LRP1 knockdown in H4 cells and human iPSC‐derived neurons completely abolished the endocytosis of monomers of all six recombinant tau isoforms, including hyperphosphorylated 2N4R (pS202/ pT205/ pS208/ pS262) and the P301L‐2N4R mutant (Rauch et al., 2020). The uptake of synthetic oligomeric 2N4R tau was also prevented, yet the internalisation of sonicated fibrils was only partially reduced (50%), suggesting that different receptors may mediate the internalisation of distinct structural forms of tau in neurons (Rauch et al., 2020). As with other LRP1 ligands (Emonard et al., 2014), two LRP1 sites mediate tau binding: the N‐terminus of tau interacts with both domain II and IV of LRP1, whereas the MTBR interacts only with domain IV (Rauch et al., 2020). The involvement of LRP1 in tau spread was tested *in vivo* in 6‐week‐old WT mice by shRNA‐mediated knockdown (Rauch et al., 2020). Tau spread was assessed using an adeno‐associated virus encoding GFP‐2A‐human 2N4R‐P301L mutant tau. This chimera undergoes self‐proteolysis at the 2A site, thereby releasing mutant tau from GFP to enable the tracking of tau propagation throughout the brain. Three weeks after injection, analysis of brain sections revealed that in mice where LRP1 was down‐regulated, tau signal was restricted to GFP‐positive neurons at the site of adeno‐associated virus injection, whereas when LRP1 was normally expressed, human tau signal was detected throughout the contralateral hippocampus and cortex. These findings support the hypothesis that LRP1 dictates tau spread (Rauch et al., 2020).

Since LRP1 has an established role in embryonic development (Kanekiyo & Bu, 2014; Mao, Xie, & Pi, 2017), these findings are particularly interesting for familial tauopathies where pathological tau may undergo LRP1‐mediated cell to cell spreading from birth. However, there is debate on the changes in LRP1 expression over time in AD. While some groups have observed a two‐fold decrease in LRP1 in AD brains compared to controls (Kang et al., 2000), others have found that LRP1 mRNA levels were increased in AD brains (Matsui et al., 2007). Interestingly, in non‐pathological aging, LRP1 decreases over time in the frontal cortex and blood–brain barrier (Kang et al., 2000; Shibata et al., 2000). Hence, future studies are necessary to determine the relative importance of LRP1 in tau uptake in aged versus young mice.

Importantly, LRP1 is ubiquitously expressed in all regions of the rodent brain (Auderset, Cullen, & Young, 2016; Bu, Maksymovitch, Nerbonne, & Schwartz, 1994), localising mainly at sites adjacent to the PSD (Nakajima et al., 2013). Since CME occurs primarily at extrasynaptic sites, it is reasonable to postulate that LRP1 could mediate receptor‐dependent endocytosis of tau at the synapse. It is worth mentioning that LRP1 participates in NMDA‐induced endocytosis of α‐amino‐3‐hydroxy‐5‐methyl‐4‐isoxazoleproprionic acid receptors (Nakajima et al., 2013), which demonstrates how physiological LRP1 processes could be hijacked by tau. Given that LRP1 is not detected in the presynaptic compartment (May et al., 2004; Nakajima et al., 2013), retrograde tau transmission is unlikely to be mediated by LRP1.

#### 4.1.2 Clathrin‐independent endocytosis of tau

##### The role of dynamin, actin and caveolin

Despite this evidence, the role of CME in tau uptake is highly debated. Pitstop 2B specifically inhibits clathrin function by blocking the binding of accessory proteins to its N‐terminal domain, which is required for CCP maturation, thus preventing CME (von Kleist et al., 2011). Upon pre‐treatment with Pitstop 2B, no change in the uptake of recombinant 2N4R tau aggregates (Wu et al., 2013) or AD brain‐derived tau oligomers (Puangmalai et al., 2020) was observed in primary neuronal cultures. Furthermore, siRNA‐mediated knockdown of clathrin heavy chain in C17.2 cells, did not affect uptake of recombinant sonicated 2N4R MTBR fibrils (Holmes et al., 2013), suggesting a role for CIE mechanisms in this process.

CIE includes several endocytic mechanisms involving alternative coat proteins, such as caveolin and flotillin (Mayor & Pagano, 2007). Interestingly, dynamin has also been found to play fundamental roles in CIE, including caveolae‐mediated internalisation and other endocytic processes involving the small GTPases, RhoA and Rac1 (Doherty & McMahon, 2009; Mayor & Pagano, 2007). CIE largely relies on lipid microdomains enriched in cholesterol and sphingolipids on the plasma membrane (Costa Verdera, Gitz‐Francois, Schiffelers, & Vader, 2017; Mulcahy, Pink, & Carter, 2014), which are known to be exploited by viral and bacterial pathogens for their internalisation (Bavari et al., 2002; Gatfield & Pieters, 2000; Schiavo & van der Goot, 2001).

Other examples of CIE include fluid‐phase endocytosis and macropinocytosis whereby large volumes of extracellular medium are internalised via a non‐selective mechanism largely dynamin‐independent (Mayor & Pagano, 2007). Macropinocytosis is a unique form of CIE mediated by actin‐dependent rearrangements of a large area of the plasma membrane leading to the formation of intracellular vacuoles spanning 0.5–5 µm, known as macropinosomes (Stillwell, 2016).

While less is known about CIE cargo selection, several probes are routinely used as CIE markers, such as HMW dextran, which is used to monitor bulk/fluid‐phase endocytosis (Ramoino et al., 2001) and TAT, a HIV‐derived peptide used to mark macropinocytosis (Kaplan, Wadia, & Dowdy, 2005).

Among CIE mechanisms, caveolin‐mediated uptake has been ruled out as a major route of tau entry at synapses, given the lack of co‐localisation between recombinant sonicated MTBR fibrils and caveolin‐1 post uptake (Holmes et al., 2013) and the inability of nystatin, which blocks the formation of caveolae, to significantly reduce uptake of human brain‐derived tau oligomers in cortical neurons (Puangmalai et al., 2020).

**FIGURE 2:**
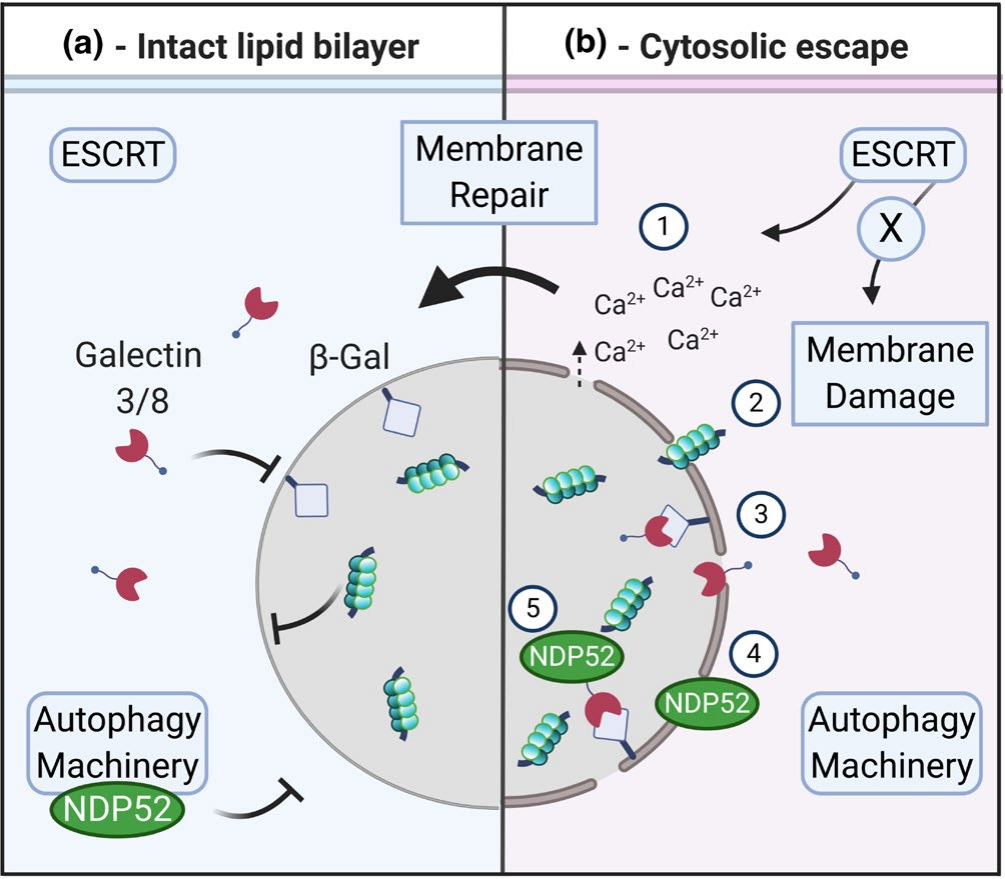
Proposed cellular response to tau‐induced endo‐lysosomal membrane damage. Following endocytosis, tau has been shown to induce membrane damage in endo‐lysosomal vesicles. (a) While the lipid bilayer is intact, components of the endosomal sorting complex required for transport (ESCRT), galectins and the autophagy machinery (marked by nuclear dot protein 52 – NDP52) are localised to the cytosol. Here, galectins are unable to access beta‐galactosides (β‐Gal), which are present together with tau, within the lumen of endo‐lysosomal compartments. (b) Tau‐induced membrane damage triggers local calcium release at the site of damage (1); If the ESCRT machinery is recruited, this leads to membrane repair, which prevents tau release. However, if this ESCRT‐mediated process fails, membrane damage will worsen and enable the escape of tau seeds and other luminal cargoes into the cytosol (2); this enables galectin3/8 access to the β‐Gal residues (3), triggering the recruitment of NDP52 (4) and the autophagy machinery to degrade the damaged organelle (5)

The role of dynamin in the CIE of tau is still controversial. Pre‐treatment of C17.2 cells with dynasore had no effect on the uptake of recombinant sonicated MTBR fibrils (Holmes et al., 2013), or the internalisation of AD tau oligomers in cortical neurons (Puangmalai et al., 2020). In contrast, Wu and colleagues demonstrated a dose‐dependent inhibition of the endocytosis of recombinant LMW tau aggregates upon dynasore incubation (Wu et al., 2013). This group also observed an 83% co‐localisation between dextran and LMW tau aggregates following uptake in neurons, inferring a bulk/fluid‐phase endocytic mechanism (Wu et al., 2013). The co‐localisation between human tau oligomers and dextran has also been found in cultured primary neurons; however, this process was dynamin‐independent (Puangmalai et al., 2020), highlighting that the CIE of tau can be both dynamin‐dependent (Figure 1b; 2) or ‐independent (Figure 1b; 3–5).

The role of actin has also been explored in tau internalisation. Phalloidin, a marker of filamentous actin, was found to decorate large organelles with a diameter of 5 µm containing recombinant sonicated MTBR fibrils in C17.2 cells. Accordingly, the actin polymerisation inhibitors cytochalasin D and latrunculin were shown to decrease MTBR fibril uptake (Holmes et al., 2013). These findings suggest that tau aggregates undergo macropinocytosis in C17.2 cells (Figure 1b; 3).

Experiments assessing the roles of actin and dynamin were also performed in human iPSC‐derived cortical neurons. Their incubation with cytochalasin D prior to addition of recombinant 2N4R‐P301S tau caused a 95% reduction of monomeric tau internalisation, yet this treatment had negligible effects on the uptake of tau aggregates (Evans et al., 2018). Treatment of iPSC‐derived cortical neurons with dynasore before the addition of recombinant monomeric tau for four hours caused an initial reduction in the number of intracellular 2N4R‐P301S puncta, which by three hours had returned to levels comparable to control conditions (Evans et al., 2018). However, when examining aggregated 2N4R‐P301S, there was a 95% reduction in the number of tau puncta, which was sustained throughout the four‐hour incubation period. This initial lag in uptake for monomeric tau suggests that dynamin‐dependent endocytosis is likely to occur within the first hours of incubation, whereas over time, slower dynamin‐independent mechanisms take over (Evans et al., 2018). As a result, Evans and colleagues have proposed a multiple entry model for monomeric and aggregated tau. Monomeric tau would undergo two modes of entry, one dependent on dynamin, which is rapid and saturable, and a second delayed mode which is dynamin‐independent but actin‐dependent and likely to proceed by macropinocytosis. Aggregated tau uptake relies on dynamin, but not on actin, suggesting that aggregated tau does not enter via macropinocytosis (Evans et al., 2018). Critically, this work did not directly address the involvement of clathrin in the dynamin‐dependent endocytic process. Future studies are therefore needed to assess whether this form of tau endocytosis in human neurons is in fact clathrin‐dependent.

Inconsistencies remain in the observed internalisation mechanisms of tau. The model proposed by Evans and colleagues (Evans et al., 2018) does not provide a conclusive mechanism for tau uptake, however, it validates previous results obtained in cultured neurons. Multiple studies have indeed provided evidence of a dynamin‐dependent tau uptake mechanism in primary neuronal cultures (Calafate et al., 2016; Evans et al., 2018; Wu et al., 2013), although, Holmes et al did not observe any role for dynamin in the uptake of tau fibrils in C17.2 cells (Holmes et al., 2013). Likewise, Puangmalai and colleagues observed no dynamin dependence for the endocytosis of human AD tau oligomers in cortical neurons (Puangmalai et al., 2020). The importance of actin for tau uptake has also been disputed using different cell‐types (Evans et al., 2018; Falcon, Noad, et al., 2018; Holmes et al., 2013). These findings highlight that both the cellular system under investigation and the origin of tau species influence tau internalisation, thus strengthening the view that different endocytic mechanisms may contribute to the uptake of distinct tau forms in different cell‐types.

##### Heparan sulphate proteoglycans

Several lines of evidence suggest that HSPGs are involved in tau internalisation. There are three main subtypes of HSPGs: transmembrane HSPGs, for example, glypicans and syndecans, which are linked to the cell membrane either via a glycosylphosphatidylinositol anchor or a transmembrane domain, respectively; secreted HSPGs, including perlecans or agrins, which are part of the extracellular matrix and serglycins, that are found in secretory vesicles of mast and hematopoietic cells (Condomitti & de Wit, 2018; Sarrazin, Lamanna, & Esko, 2011). While HPSGs have a variety of structures, they all contain one or more heparan sulphate (HS) chain covalently linked to the core protein domain. HS chains are glycosaminoglycans formed of repeating disaccharide units typically made up of glucuronic acid (GlcA) and N‐acetyl‐D‐glucosamine (GlcNAc) bearing multiple modifications. Importantly, the majority of HPSG ligand interaction occurs on the HS chains (Sarrazin et al., 2011).

HSPGs were identified as a cellular receptor for the prion protein, growth factors and neurotropic viruses (Horonchik et al., 2005); accordingly, HSPG inhibitors and heparan mimetics reduce the extracellular binding and uptake of prions (Horonchik et al., 2005; Schonberger et al., 2003). It has also been shown that tau binds heparin, a densely sulphated form of HS, through both its N‐terminus and the MTBR, and this interaction can initiate tau aggregation (Goedert et al., 1996).

In 2013, HSPGs were proposed to mediate the internalisation of tau in cell lines and primary neurons both *in vitro* and *in vivo*. Recombinant sonicated tau MTBR fibrils were found to co‐localise with TAT (Holmes et al., 2013), a macropinocytosis marker that requires HSPGs for uptake (Tyagi, Rusnati, Presta, & Giacca, 2001); interestingly, the TAT signal encapsulated tau puncta, supporting the hypothesis that tau fibrils are internalised within macropinosomes upon interaction with HSPGs (Holmes et al., 2013) (Figure 1b; 3). While TAT is a marker of HSPG‐mediated macropinocytosis, it is also used to aid the direct translocation of peptides across biological membranes (Takeuchi & Futaki, 2016), and so caution must be taken in interpreting these data.

Contrasting results have been obtained while investigating the involvement of HSPGs in the uptake of recombinant monomeric tau. Intracellular MTBR‐tau signal was observed eight hours post‐incubation, despite the absence of glycosaminoglycans on the cell surface of pgsA‐745 cells (Michel et al., 2014). However, blocking HSPGs inhibits fibrillar tau uptake after three hours of incubation (Holmes et al., 2013), suggesting that HSPG‐independent mechanisms may drive monomeric tau internalisation at later time‐points in pgsA‐745 cells.

Given the fundamental structural differences between tau aggregates generated *in vitro* using heparin, and those extracted from human diseased brains (Zhang et al., 2019), in addition to the evidence of disease‐specific tau strains, it is likely that unique structural features influence tau uptake. To address this, the internalisation mechanism of tau oligomers obtained from postmortem human brain extracts of AD, dementia with Lewy bodies and PSP was investigated (Puangmalai et al., 2020). These oligomers were analyzed with atomic force microscopy and size‐exclusion chromatography and found to range between 270–670 kDa. Convincingly, all three pathological tau oligomers were endocytosed by primary cortical neurons, although to varying degrees (Puangmalai et al., 2020). Having excluded the role of CME, the authors administered heparin as a competitive inhibitor for tau uptake and this treatment reduced internalisation of all tau oligomers. Whilst AD tau was highly affected by HSPG inhibition, PSP tau was only weakly reduced (Puangmalai et al., 2020). As a result, HSPG‐mediated macropinocytosis seems to be the predominant internalisation mechanism for AD oligomers, whereas PSP tau is likely to exploit multiple endocytic pathways.

Extensive work has identified the importance of specific sites of HSPG sulphation for tau uptake. Along the disaccharide unit of the HS chain, there are a number of different sulphation sites at specific nitrogen and oxygen moieties (Sarrazin et al., 2011). Pharmacological inhibition of HSPG sulphation reduced the ability of both MTBR and full length sonicated tau fibrils to bind the plasma membrane and be internalised (Holmes et al., 2013). In a further effort to identify specific HSPG modifications involved in tau uptake, 2‐O, 6‐O and N‐desulphated heparin derivatives were tested for their ability to bind tau *in vitro*; recombinant 2N4R fibrils bound heparin and De‐2‐O, but weakly interacted with the De‐6‐O or De‐*N* (Stopschinski et al., 2018). These results were confirmed in C17.2 cells, where pre‐incubation of recombinant sonicated tau fibrils with heparin and De‐2‐O strongly inhibited tau uptake, whilst incubation with De‐6‐O and De‐N were largely ineffective (Stopschinski et al., 2018). Other groups have independently identified 6‐O sulphation but not N‐sulphation to be important for the uptake of recombinant monomeric and oligomeric 2N4R tau in H4 cells and human IPSC‐derived neurons (Rauch et al., 2018). The inconsistent results may be because of the use of different tau species between the two groups resulting in differential sensitivities to HSPG sulphation, thus influencing the uptake dynamics.

Large‐scale CRISPR genetic screens have identified multiple genes of the HSPG biosynthetic pathway to be involved in tau uptake, such as HS6ST2, which mediates 6‐O sulphation of HSPGs (Stopschinski et al., 2018), and Ext1 and Ext2, two enzymes involved in nascent HS chain polymerisation by catalysing the addition of GlcA and GlcNAc (Sarrazin et al., 2011). Knockdown of Ext1/Ext2 has been shown to inhibit uptake of recombinant monomeric and fibrillar 2N4R tau and of human tau oligomers extracted from both AD and dementia with Lewy bodies brains in HEK293 cells, primary cortical and hippocampal neurons as well as human iPSC derived neurons (Holmes et al., 2013; Puangmalai et al., 2020; Rauch et al., 2018; Stopschinski et al., 2018).

The important role of HSPGs in tau dynamics has been validated both ex vivo and *in vivo*. Preincubation of recombinant monomeric 2N4R tau with heparin prevented its uptake in acute adult mouse brain slices, whereas 6‐O desulphated heparin did not (Rauch et al., 2018). Furthermore, co‐injection of full‐length sonicated 2N4R fibrils with a heparin mimetic (F6) in 5‐month‐old WT mice, caused a 30% reduction of tau signal compared to vehicle‐injected controls, confirming the importance of HSPGs in tau uptake *in vivo* (Holmes et al., 2013).

HSPGs could also participate in the trans‐synaptic spread of tau *in vivo* given their localisation at pre‐ and post‐synaptic sites. HS chains are found on different adhesion proteins and play a crucial role in synapse specification (Condomitti & de Wit, 2018; Zhang et al., 2018). The 6‐O sulphation of HS chains, which contributes to tau binding, can be removed by two endosulphatases encoded by Sulf1 and Sulf2. Sulf2 is present in most brain regions in adult mice, including the hippocampus, whereas Sulf1 is mostly expressed during development (Kalus et al., 2009). Sulf1 knockout specifically affected dendritic spine density and long term potentiation (LTP), suggesting that a tight regulation of 6‐O sulphation is required at the synapse for optimal functioning (Kalus et al., 2009). Given the vast number of proteins that can be bound by HS chains, and the paucity of tools to localise different HS groups, it is difficult to precisely assess where the interaction between HS and tau on the neuronal plasma membrane takes place.

Syndecans belong to one of three subtypes of HSPGs and consist of four members (syndecan 1–4) (Condomitti & de Wit, 2018). A significant elevation of the level of all syndecans except syndecan‐1 was detected in post‐mortem AD brains compared to controls (Liu et al., 2016; Lorente‐Gea et al., 2020), suggesting their involvement in AD. Indeed, increased expression of syndecans 2–4 has been associated with neurodegeneration vulnerability in AD (Grothe et al., 2018). Syndecans are known to undergo ligand‐induced CIE (Condomitti & de Wit, 2018), thus proving to be potentially involved in tau internalisation. In K562 cells which do not express HSPGs, recombinant sonicated 2N4R fibrils displayed negligible uptake, which was restored following transfection with all four individual syndecans, with the greatest effect seen with the neuronal syndecan‐3 (Hudák et al., 2019). Transferrin internalisation was largely unaffected by syndecan expression, thus ruling out CME in this process.

Syndecan‐mediated uptake appears specific to fibrillar tau (Hudák et al., 2019). Interestingly, the authors observed that after incubation of monomeric tau in the presence of syndecan 2–4, tau fibrils would first appear on the cell‐surface before internalisation. This suggests that tau receptors may also contribute to tau aggregation thus facilitating uptake of seed‐competent fibrillar tau. Co‐localisation and immunoprecipitation experiments have revealed a lipid microdomain‐dependent pathway of internalisation for tau fibrils involving syndecans and flotillins, membrane proteins associated to these lipid assemblies (Hudák et al., 2019) (Figure 1b; 4). Crucially, flotillins and syndecans localise both at pre‐ and post‐synaptic sites, where they have well‐known roles in synaptic transmission (Bodrikov, Pauschert, Kochlamazashvili, & Stuermer, 2017; Francesconi, Kumari, & Zukin, 2009; Hering, Lin, & Sheng, 2003; Suzuki et al., 2001; Swanwick, Shapiro, Vicini, & Wenthold, 2010), thus possibly contributing to the trans‐synaptic transfer of tau.

Syndecan‐3, which displays the strongest effect on tau internalisation (Hudák et al., 2019), is enriched in neurons with roles in synaptic plasticity (Hsueh, 2006; Lauri et al., 1999). LTP increases syndecan‐3 levels and promotes its pre‐synaptic relocalisation from axonal sites (Lauri et al., 1999). Thus, syndecan‐3 redistribution during synaptic plasticity could enhance tau binding and uptake at synapses. In contrast, syndecan‐2 localises at dendrites where it participates in the formation and maturation of dendritic spines (Hsueh & Sheng, 1999) via modulation of actin dynamics (Lin, Lei, Hong, & Hsueh, 2007). Syndecan‐2 also appears to be a good candidate for the trans‐synaptic transfer of tau given its post‐synaptic localisation and its ability to modulate actin, an important component of endocytosis. Syndecan‐1 and −4 are unlikely to be major players in inter‐neuronal tau spread because of their restricted localisation; syndecan‐1 is mainly expressed in cerebellar granule neurons, which are largely spared in tauopathies, with the exception of PSP (Braak, Braak, Bohl, & Lang, 1989; Irwin, 2016) and syndecan‐ 4 mainly localises in glial cells (Hsueh & Sheng, 1999).

Syndecans have been shown to promote 6‐O sulphation on HS chains of HSPGs (Corti et al., 2019), which appears to specifically mediate tau uptake (Rauch et al., 2018; Stopschinski et al., 2018). Interestingly, HSPGs bind LRP1 and have been involved in its internalisation (Kanekiyo et al., 2011). Furthermore, some LRP1 ligands are able to bind HSPGs to facilitate LRP1‐mediated endocytosis (Bres & Faissner, 2019). However, these interactions have not been investigated in the context of tau uptake, in particular with a focus on syndecans. HSPGs are known to be involved in Aβ, α‐synuclein and prion uptake (Horonchik et al., 2005; Kanekiyo & Bu, 2014; Kanekiyo et al., 2011; Sandwall et al., 2010; Schonberger et al., 2003; Stopschinski et al., 2018) and so their involvement could underlie a pathological mechanism shared between amyloid disorders.

The interaction between pathological proteins including tau, Aβ, α‐synuclein and prion, is an active area of study (Busche & Hyman, 2020), given their co‐occurrence in protein aggregates observed in post‐mortem brain samples of patients diagnosed with different neurodegenerative diseases (Spires‐Jones, Attems, & Thal, 2017). Recent work demonstrates mechanistic crosstalk between these proteins also in transcellular spread. One example includes the interaction between tau and Aβ; cellular pre‐incubation of recombinant oligomeric Aβ was shown to promote tau uptake in HEK293 cells, SH‐SY5Y cells and primary hippocampal neurons (Shin et al., 2019). This phenomenon was observed across a range of tau forms including recombinant full length and MTBR 2N4R tau and tau aggregates obtained from the PS19 tauopathy mouse model, which over‐express human 1N4R‐P301S. This effect was in‐part dependent on HSPGs, as increasing doses of heparin reduced the Aβ‐mediated increase of tau uptake in HEK293 biosensor cells (Shin et al., 2019).

A similar relationship has been reported for prion protein and tau. De Cecco and colleagues have shown that recombinant sonicated MTBR fibrils were able to bind soluble prion protein *in vitro* (De Cecco et al., 2020). Interestingly, both CRISPR‐mediated knockdown and antibody‐mediated block of prion protein caused a significant reduction in the uptake of MTBR fibrils, supporting the hypothesis that prion protein facilitates tau uptake (De Cecco et al., 2020).

The interplay between tau and other proteins involved in neurodegeneration known to spread in a prion‐like manner needs to be further investigated to identify which mechanisms are shared with tau and which are specific for different aggregation‐prone proteins.

### 4.2 Neuronal entry of extracellular vesicles containing tau

Much of the work discussed so far focuses on uptake mechanisms for free tau; however, several lines of evidence support the involvement of EVs in extracellular tau dynamics (Lee et al., 2019). In particular, exosomes containing tau have been found in both CSF and blood samples of AD patients (Guix et al., 2018), suggesting a role for EVs in AD pathology. In EVs isolated from either cultured neurons (Wang et al., 2017) or mouse brain (Polanco et al., 2016), tau is present in their lumen, as shown by its resistance to degradation upon protease treatment.

Studies examining EV internalisation demonstrated that this process is time‐ and temperature‐dependent (Costa Verdera et al., 2017), suggesting an active endocytic mechanism, the details of which are not completely clarified. There has been evidence that Pitstop 2B can reduce uptake of exosome‐like EVs (Horibe, Tanahashi, Kawauchi, Murakami, & Rikitake, 2018) (Figure 1b; 1); however, treatment with chlorpromazine, which prevents clathrin binding to the membrane, did not block EV uptake (Costa Verdera et al., 2017). Steric restraints are likely to play a role in EV uptake. Exosome diameters range from 50 to 150 nm and ectosomes can be up to 1 µm (Mathieu et al., 2019), whereas the average diameter of CCVs is around 100 nm (Pearse, 1976), making it unlikely for EVs to be predominantly internalised via CME. Indeed, accumulating evidence indicates that EVs are internalised via multiple CIE routes, including lipid microdomain‐mediated uptake, as demonstrated by the dose–dependent decrease of EV internalisation caused by inhibiting cholesterol synthesis (Costa Verdera et al., 2017) (Figure 1b; 4–5). Macropinocytosis has also been considered due to the large size of macropinosomes (Stillwell, 2016) and the pharmacological inhibition by EIPA, which reduces EV uptake (Costa Verdera et al., 2017) (Figure 1b; 3). However, direct evidence for the uptake mechanism of tau‐containing EVs is currently lacking.

Exosome‐enriched EVs can be isolated from AD mouse models and human patient brains and characterised by size distribution and the presence of established exosomal markers. Exosome‐enriched EVs were extracted from rTg4510 mice brain and incubated with HEK cells expressing CFP/YFP MTBR‐P301S tau, a FRET biosensor for tau aggregation. A weak but significant FRET signal was detected after 72 hr of EV treatment, confirming that tau‐containing EVs are internalised and release their contents into the cytosol to seed aggregation of MTBR‐P301S tau (Polanco et al., 2016). Exosome‐like EVs from rTg4510 mice were also found inside MVBs in the soma, dendrites and axon termini of hippocampal neurons as detected by electron microscopy (Polanco et al., 2018).

Recent work by Ruan and colleagues investigated exosome‐enriched EVs isolated from post‐mortem human brain from both control subjects (CTRL‐EV) and AD patients at prodromal (pAD‐EV) or symptomatic stage (AD‐EV) (Ruan et al., 2020). In primary neurons, AD‐EVs exhibited higher uptake levels and transfer into the cytosol compared to both pAD‐EVs and CTRL‐EVs (Ruan et al., 2020). These results suggest that EVs isolated from different pathological conditions may display fundamental differences (e.g. specific surface receptors), which may dictate the efficiency of uptake. To assess the propagation of EVs *in vivo*, 18‐month‐old WT mice were injected with AD‐EVs in the outer molecular layer of the dentate gyrus. Tau pathology was observed at the site of injection and across the hippocampus, including the CA1 and CA3 regions, 4.5 months after injection, suggesting that EVs had been internalised and pathology had spread (Ruan et al., 2020). When equal amounts of free tau oligomers and fibrils were extracted from the same post‐mortem AD brain sample and injected into WT mice, there was no widespread tau pathology, despite strong signal seen at the injection site (Ruan et al., 2020). This is the first study demonstrating enhanced propagation of human post‐mortem derived EV‐tau versus free tau (Ruan et al., 2020).

Overall, many mechanistic gaps are still present in our understanding of EV physiology, which are key to elucidate tau transmission. Given EV cellular uptake is not clear, it is also unknown whether release of EV cargoes into the cytosol occurs via direct fusion with the plasma membrane from the extracellular space, or via endosomal escape, which could involve fusion with the endosomal membrane (Mathieu et al., 2019). With regards to tau, it is unclear if direct interaction of tau with phospholipids could facilitate these mechanisms. Interestingly, several tau isoforms have been shown to interact with phospholipids. Tau‐phospholipid complexes obtained by mixing brain phosphatidylserine liposomes with full length recombinant tau were recruited to the neuronal plasma membrane and taken up after 24 hr (Ait‐Bouziad et al., 2017). When compared to free recombinant sonicated fibrils, the interaction between tau and lipids appeared to enhance tau endocytosis. Overall, understanding how tau interacts with lipid bilayers is important for its neuronal internalisation and for its release from the EV lumen into the cytosol, as discussed below.

### 4.3 Nanotubes and tau transfer

While endocytosis has dominated the field of tau uptake and transmission, tunnelling nanotubes (TNTs) have recently emerged as an alternative mechanism for intercellular tau transfer. TNTs are elongated actin‐containing structures ranging from 0.1 to 100 µm in length, which protrude from cells to form physical connections lasting for minutes up to hours (Sartori‐Rupp et al., 2019). Under physiological conditions, TNTs have been reported to traffic mitochondria and other cargoes (Sartori‐Rupp et al., 2019). Recently, TNTs have been implicated in neurodegenerative diseases, since pathological proteins, such as prion and Aβ, promote TNT formation and exploit these structures for their transfer between cells (Gousset et al., 2009; Wang, Cui, Sun, & Zhang, 2011). Similarly, TNT formation is induced upon five‐minute incubation of primary cortical neurons with recombinant sonicated 1N4R tau fibrils. These TNTs contained both exogenous human 1N4R tau as well as endogenous murine tau (Tardivel et al., 2016). Following 6 hr incubation, fluorescently labelled 1N4R fibrils were shown to travel from the donor to the receiving neuron through TNTs (Tardivel et al., 2016). In neuronal cell lines, the average speed of tau fibrils transport in TNTs was 2.8 ± 2.0 μm/min (Tardivel et al., 2016), which is comparable with that observed for actin‐based processes. While recombinant tau fibrils were shown to transfer across cells via TNTs (Figure 1b; 6), the tau species undergoing cell‐to‐cell transmission via TNTs *in vivo* is currently unknown.

A recent study examined the structure of TNTs using a wide range of high‐resolution microscopy approaches. This enabled the detection of vesicles within TNTs, including single‐membrane organelles and MVB‐like compartments with diameters ranging between 50 and 200 nm (Sartori‐Rupp et al., 2019). Given the complex nature of tau transmission, free tau as well as membrane encapsulated tau could be transported via TNTs to mediate propagation of tau pathology (Figure 1b; 6).

### 4.4 Release of internalised tau in the cytosol

As described in detail above, independent evidence supports the view that tau enters neurons mostly by endocytosis, implying that pathological tau must cross the endosomal membrane to reach the cytosol and act as a pathological seed. However, most of the work presented in previous sections does not address whether tau localises in vesicles or in the cytosol after internalisation. This is mostly because of technical challenges, which do not allow reliable discrimination between these two intracellular locations. In addition, the complete process of internalisation and release is relatively slow and thus difficult to follow in real‐time. For example, Wu and colleagues have shown that tau secreted by rTg4510 neurons was observed after few hours as a punctate signal in ‘recipient’ WT neurons, suggestive of its presence in vesicular compartments. Only after six days, the signal shifted into a diffuse staining, indicating that tau had entered into the cytosol (Wu et al., 2016).

As a result, the mechanism exploited by tau to escape the endosomal lumen and reach the cytosol has not been investigated in detail, and when addressed, the evidence provided has often been indirect. *In vitro* experiments with recombinant tau and artificial vesicles suggested that both recombinant monomeric and fibrillar tau can disrupt vesicles, possibly by extracting phospholipids from lipid bilayers (Ait‐Bouziad et al., 2017; Flavin et al., 2017). However, it is unclear where and when this process takes place. Internalised tau travels through the endo‐lysosomal pathway as shown by co‐localisation with markers of early endosomes, such as Rab5 (Calafate et al., 2016; Falcon, Noad, et al., 2018; Puangmalai et al., 2020; Wu et al., 2013) and EEA1 (Ait‐Bouziad et al., 2017), late endosomes (Rab7; (Chen et al., 2019; Falcon, Noad, et al., 2018)) and lysosomes (LAMP1 and LAMP2; (Chen et al., 2019; Puangmalai et al., 2020; Wu et al., 2013)) (Figure 1c). These results were observed for monomeric tau, heparin‐assembled fibrils, tau aggregates isolated from human brains, cell‐secreted tau and tau‐phospholipid complexes. Accordingly, recombinant tau, both in its monomeric and heparin‐induced aggregated forms, has also been shown to localize in early and late endosomes/lysosomes in human iPSC‐derived excitatory neurons (Evans et al., 2018), suggesting that this is a conserved mechanism. In contrast, recent work has shown that the post‐endocytic processing of tau aggregates obtained from AD and PSP brains is different (Puangmalai et al., 2020). In particular, PSP aggregates appeared to be internalised bypassing early endosomes, but still reached lysosomes when HSPG‐mediated uptake was inhibited, suggesting that different disease‐specific tau aggregates could follow diverse uptake mechanisms, leading to different intracellular fates.

The escape of tau from intracellular vesicles through a membrane damaging action has been inferred mostly by changes in the localisation pattern of galectins (Figure 2). Galectins are a family of secreted lectins that bind to extracellular carbohydrates, in particular β‐galactosides (Johannes, Jacob, & Leffler, 2018). However, recent data have shown that galectins also function as ‘danger receptors’ for endo‐lysosomal damage (Thurston, Wandel, von Muhlinen, Foeglein, & Randow, 2012). Upon endocytosis, β‐galactosides are hidden in the lumen of the endo‐lysosomes; however, membrane damage of these vesicles exposes these carbohydrates to the cytosolic environment where galectins are also found. This leads to a rapid rise in the concentration of galectins at damaged endo‐lysosomes with a subsequent recruitment of autophagy complexes for rapid organelle removal (Johannes et al., 2018). Interestingly, recruitment of galectin 3 and 8 to tau‐containing endo‐lysosomes has been observed 24 hr after tau incubation in cell lines and cultured primary neurons (Calafate et al., 2016; Chen et al., 2019; Falcon, Noad, et al., 2018; Flavin et al., 2017) (Figure 2). In most cases, heparin‐induced recombinant fibrils obtained from either WT 0N4R, 1N3R and 1N4R or mutant (0N4R‐P301S) (Falcon, Noad, et al., 2018) tau were used, although similar observations were made with tau secreted by seeded HEK cells over‐expressing 2N4R‐P301L‐GFP mutant tau (Calafate et al., 2016). These findings indicate that endo‐lysosomal damage is induced by tau aggregates and may lead to the release of pathological tau seeds into the cytosol. As mentioned above, the recruitment of galectins triggers the activation of the autophagy machinery (Figure 2), as observed in cell lines where galectin 8 puncta co‐localised with tau seeds and the autophagy marker nuclear dot protein 52 (NDP52) (Falcon, Noad, et al., 2018), which is known to bind galectin 8 (Thurston et al., 2012). Accordingly, galectin 8 down‐regulation increased seeded aggregation in HeLa cells, suggesting an increased escape of tau seeds (Falcon, Noad, et al., 2018).

The endosomal sorting complex required for transport (ESCRT) has also been implicated in the process of tau‐induced endo‐lysosomal membrane damage (Chen et al., 2019) (Figure 2). The ESCRT system is composed of several protein complexes enabling multiple pathways, such as vesicle budding and membrane repair (Hurley, 2015). This complex is recruited at sites of organelle damage, where it is activated by local calcium release (Hurley, 2015; Skowyra, Schlesinger, Naismith, & Hanson, 2018). Although the exact mechanism of membrane repair is not yet clear, it has been suggested that the ESCRT complex promotes budding and severing of the damaged membrane. Alternatively, it may assist in the packaging of the damaged organelle into a new vesicle (Hurley, 2015; Skowyra et al., 2018).

At least three components of the ESCRT machinery, CHMP2A, CHMP2B and CHMP6, contribute to the repair of endo‐lysosomal membrane damage induced by tau. Their down‐regulation was found to increase tau aggregation in HEK cells upon seeding with recombinant 0N4R‐P301S tau fibrils or phosphotungstate‐insoluble material isolated from AD brains (Chen et al., 2019). This phenotype was mimicked by application of L‐leucyl‐L‐leucine‐O‐methyl ester (LLOME), a lysosomotropic agent able to accumulate in acidified organelles and be processed by cathepsin C, generating membrane damaging polymers (Maejima et al., 2013; Thiele & Lipsky, 1990). Interestingly, the escape of tau from endo‐lysosomal compartments appears to be controlled and limited by the ESCRT machinery, which is recruited early on in the repair process, at the appearance of small holes (Skowyra et al., 2018). If the ESCRT machinery fails to repair the damage, the membrane gaps would increase in size, allowing galectins to be recruited and activate autophagy in a final attempt to stop the release of endo‐lysosomal content (Skowyra et al., 2018) (Figure 2). Although appealing, this sequential chain of events has not been yet demonstrated in neurons nor upon tau‐induced membrane damage. Nevertheless, pathways involved in damage detection and membrane repair might have a protective and limiting effect on the propagation of tau pathology and could therefore present opportunities for therapeutic intervention in tauopathies.

Interestingly, both tau and Aβ exposure can induce endosomal and lysosomal abnormalities (Peric & Annaert, 2015; Puangmalai et al., 2020; Wang et al., 2009; Yang, Chandswangbhuvana, Margol, & Glabe, 1998) which in turn would increase the residing time of tau seeds in these compartments. This could promote their aggregation (Michel et al., 2014) and thus their membrane‐damaging action, and/or could enhance incomplete tau processing with the generation of fragments with increased toxicity and seeding potency (Khurana et al., 2010; Meduri et al., 2016; Wang et al., 2009). In addition, the ability of tau fibrils to disrupt vesicles upon binding to their external membrane leaflet (Ait‐Bouziad et al., 2017; Flavin et al., 2017), suggests a potential self‐sustaining mechanism where cytosolic pathological tau could promote liberation of additional tau seeds from intracellular vesicles further enhancing pathology.

In summary, the biological mechanism of tau release into the cytosol is still relatively unclear; further research is therefore required to gain a more in‐depth understanding of this process in both physiological and pathological states.

## 5 MIND THE GAP: FUTURE PERSPECTIVES FOR THE TAU FIELD

As described above, tau internalisation is a critical step for the prion‐like spread of tau pathology. In this review, we have highlighted recent evidence demonstrating that tau enters neurons from the extracellular milieu, a phenomenon that has been confirmed both in mouse models and in human iPSC‐derived neurons (Table 2). There is, however, still debate as to which species of tau transfers between cells and eventually acts as a pathological seed. Within the extracellular space, tau is either free or within EVs, with the former considered the predominant species. Interestingly, vesicular tau detected in AD CSF is seed‐competent, suggesting that tau within EVs is also pathologically relevant (Wang et al., 2017). Despite the low abundance of EVs in the extracellular space, small amounts of misfolded tau may be sufficient to support pathological tau propagation, given the self‐sustaining nature of this process.

Pathological tau exists along a spectrum of aggregated states, with monomers at one end, fibrils at the other, and several oligomeric intermediates completing this continuum (Goedert, Eisenberg, & Crowther, 2017; Wang & Mandelkow, 2016). While most tau species have been tested for uptake, fully formed fibrils are very large entities spanning hundreds of nanometres (Kidd, 1963) and are thus unlikely to be released or taken up by cells in an intact form (Rauch et al., 2018). When used in internalisation assays, tau fibrils are routinely broken down via sonication. Although several tau species can effectively enter neurons, aggregates seem to be taken up more efficiently, compared to monomers (Frost et al., 2009; Jackson et al., 2016; Wu et al., 2013). In this regard, some authors have suggested that trimeric tau is the minimal unit required for endocytosis (Mirbaha, Holmes, Sanders, Bieschke, & Diamond, 2015). Recent studies exploring the properties of tau isolated from CSF and ventricular fluid from post‐mortem AD brains have suggested that HMW (600–700 kDa and 10–20 nm) globular tau aggregates have the greatest seeding capacity, and are more readily internalised compared to other forms (Takeda et al., 2015, 2016). Interestingly, aggregate size per se was not sufficient to enhance uptake, as recombinant tau aggregates of similar dimension failed to be internalised (Takeda et al., 2015).

Tau found in the extracellular media or CSF of AD patients presents several different modifications (Table 1), which might impact its transmission properties. One example is proteolytic processing: whilst many fragments of tau have been identified (Quinn et al., 2018; Vogels et al., 2020), the relevance of tau cleavage for cell internalisation is still unclear. Although several N‐terminal fragments lacking the MTBR domain are detected in AD patients CSF (Blennow et al., 2020; Cicognola et al., 2019) and are endocytosed by neurons (Rauch et al., 2020), it is assumed that only fragments containing the MTBR would propagate pathology, since motifs within this region are known to be critical for fibril formation (Li & Lee, 2006). Since monomeric and aggregated forms of the MTBR alone can also be internalised (Holmes et al., 2013; Rauch et al., 2020), future work is needed to establish whether N‐ and C‐terminal truncations affect the efficiency of tau uptake and its productive cytoplasmic transfer in neurons.

The role of phosphorylation in tau uptake is also not well understood. Tau hyperphosphorylation is generally accepted to be an early sign of pathology since it can decrease tau affinity for microtubules, thus promoting self‐aggregation (Wang & Mandelkow, 2016). However, some studies have shown that extracellular tau is hypo‐phosphorylated (see *Tau species in extracellular milieu*), whilst others have shown increased phosphorylation at specific sites (S199, S396, S404) in seed‐competent tau obtained from AD brain extracts or CSF (Takeda et al., 2015, 2016). Of note, dephosphorylation of these sites was found to reduce tau uptake (Takeda et al., 2015). Interestingly, a tau phosphomimetic mutant with 14 serines and threonines (including those aforementioned) mutated to glutamate (E14) (Hoover et al., 2010), was shown to transfer between neurons in MFCs (Hallinan et al., 2019). Exosomes also contain seed‐competent phosphorylated tau, with modifications at T181 and S396 reported in multiple studies (Crotti et al., 2019; Fiandaca et al., 2015; Wang et al., 2017). In conclusion, although PTMs do not appear necessary for tau uptake since recombinant tau produced in bacteria can also enter neurons (Takeda et al., 2015), they may modulate uptake dynamics.

Interestingly, physiological tau can also be found in the extracellular media of human iPSCs‐derived neurons (Chai et al., 2012) and human CSF from healthy subjects. In addition, recombinant WT tau can also enter neurons as previously discussed. This supports the hypothesis that tau can move from neuron to neuron also in physiological conditions. Whether this is an unconventional signalling pathway or just a waste removal mechanism remains unclear. It seems safe to assume that physiological tau would spread as a monomer, since formation of tau aggregates is believed to be a pathological event. However, some form of self‐interaction of tau has been recently shown to exist during microtubule binding and stabilization in physiological conditions (Siahaan et al., 2019; Tan et al., 2019).

The mechanism(s) exploited by tau to enter neurons remain controversial. As described above, the complexity and interdependence of different endocytic pathways suggest that multiple routes for tau internalisation coexist. It is thus highly likely that both CME and CIE mediate tau endocytosis and the importance of these pathways may vary in different cell types and/or for different tau species (Evans et al., 2018; Rauch et al., 2020). This is an important point to consider as tau aggregates are now recognized to have distinct structures and post‐translational modifications in different tauopathies (Arakhamia et al., 2020; Falcon, Zhang, Murzin, et al., 2018; Falcon, Zhang, Schweighauser, et al., 2018; Falcon et al., 2019; Fitzpatrick et al., 2017) and thus may follow distinct pathways of internalisation, as demonstrated by the finding that in cultured neurons, HSPGs mediate the uptake of tau aggregates obtained from AD, but not PSP, brains (Puangmalai et al., 2020). Crucially, many studies presented in this review took advantage of the ability of recombinant tau to self‐aggregate *in vitro*; however, recent work has shown that these aggregates display structures that are not observed in tauopathies (Zhang et al., 2019). Therefore, future investigations should focus on monitoring endocytosis of tau forms that better represent the human pathology.

In addition, a differential propensity for tau uptake and/or for its cytosolic release among diverse neuronal populations might in part explain the selective vulnerability hypothesis, which postulates that specific subtypes of neurons are more prone to degeneration than others (Forrest, Kril, & Halliday, 2019; Fu, Hardy, & Duff, 2018). Alternatively, specific neuronal circuits could present activity patterns enhancing specific aspects of membrane dynamics that promote tau internalisation.

As addressed throughout the review, most of the internalisation mechanisms have been shown to occur at synapses. In addition, tau aggregates obtained from AD brain extracts have been shown to localise at the post‐synaptic compartment prior to internalisation (Puangmalai et al., 2020). However, to the best of our knowledge, direct evidence of tau endocytosis at the synapse is still lacking, highlighting the urgent need for ultrastructural experiments to test endocytic mechanisms driving tau uptake at synaptic sites. Different lines of evidence converge on the importance of HSPGs, such as syndecans, and LRP1 for tau endocytosis. Interestingly, HSPGs have been found also to promote tau secretion (Katsinelos et al., 2018; Merezhko et al., 2018). As HSPGs could be involved in both CME and CIE, and are localised at synapses, they may represent hotspots for tau pathology propagation.

After internalisation, tau appears to follow a classic endocytic pathway, being found associated to early and late endosomes and to lysosomes. The acidic pH of endo‐lysosomal organelles and the presence of specific proteases may modify tau both via limited proteolysis (Khurana et al., 2010; Meduri et al., 2016; Wang et al., 2009) or even enhancing aggregation (Michel et al., 2014), thus promoting tau‐seed production. The mechanisms through which tau escapes from these compartments into the cytosol are not well defined; to date, membrane damage is the only proposed mechanism, with tau oligomers suggested to be more effective in permeabilising lipid bilayers than monomers or large fibrils (Ait‐Bouziad et al., 2017; Flach et al., 2012). Furthermore, lipid binding may also promote pathological conformational changes of tau (Ait‐Bouziad et al., 2017; Flach et al., 2012), and thus represents an area that should be further explored, as it could influence multiple steps of tau spread. Since direct translocation through membranes has been suggested as a possible secretion mechanism (Katsinelos et al., 2018; Merezhko et al., 2018), future work should explore whether this may occur during the cytosolic translocation of tau.

In conclusion, although tau intercellular transfer is now widely accepted to play a significant role in tauopathies, the mechanisms controlling tau secretion, uptake and cytosolic exit within an interconnected neuronal network, are still poorly understood and lack essential molecular insights. Blocking the spread of tau pathology represents an attractive target to slow disease progression in AD and tauopathies. Consequently, therapeutic agents acting on these pathways are actively being investigated. Existing treatments currently in clinical trial, such as passive or active anti tau immunotherapies designed to lower tau levels or reduce hyperphosphorylation (reviewed in (Congdon & Sigurdsson, 2018; Jadhav et al., 2019), may also act by sequestering free tau during its transcellular phase to avoid neuronal uptake. In addition, molecules identified to function as ‘tau receptors’ on the neuronal plasma membrane, such as muscarinic receptors, LRP1 or sulphated HSPGs, could be targeted by approaches designed to lower their levels and by small molecules or antibodies to reduce tau binding. Alternatively, identifying intracellular proteins that participate in the endocytosis or cytosolic release of tau might provide additional targets for both gene therapy and small molecule‐based therapies.

Further interdisciplinary research exploiting innovative technologies is needed to clarify the machinery controlling tau internalization, thus paving the way for the discovery of therapeutic targets to effectively treat human tauopathies.

## CONFLICT OF INTEREST

The authors have no competing interest to declare.
